# Chemotherapy in synergy with innate immune agonists enhances T cell priming for checkpoint inhibitor treatment in pancreatic cancer

**DOI:** 10.1186/s40364-024-00721-7

**Published:** 2025-01-27

**Authors:** Nan Niu, Keyu Li, Junke Wang, Vanessa Funes, Birginia Espinoza, Pan Li, Jianxin Wang, Melissa Lyman, Mengni He, Brian Herbst, Michael Wichroski, Ruslan Novosiadly, Sami Shoucair, Yiping Mou, Lei Zheng

**Affiliations:** 1 Department of Oncology and the Sidney Kimmel Comprehensive Cancer Center, Johns Hopkins University School of Medicine, Baltimore, MD 21287 USA; 2 Department of Gastrointestinal Surgery, The First Affiliated Hospital of Zhejiang Chinese Medical University (Zhejiang Provincial Hospital of Chinese Medicine), Hangzhou, Zhejiang 310003 China; 3 The Pancreatic Cancer Precision Medicine Center of Excellence Program, Johns Hopkins University School of Medicine, Baltimore, MD 21287 USA; 4 The Bloomberg Kimmel Institute for Cancer Immunotherapy, Johns Hopkins University School of Medicine, Baltimore, MD 21287 USA; 5 Division of Pancreatic Surgery, Department of General Surgery, West China Hospital, Sichuan University, Chengdu, Sichuan 610041 China; 6 The Multidisciplinary Gastrointestinal Cancer Laboratories Program, the Sidney Kimmel Comprehensive Cancer Center, Johns Hopkins University School of Medicine, Baltimore, MD 21287 USA; 7 The First-affiliated Hospital of Zhejiang University, Hangzhou, Zhejiang China; 8 Bristol Myers Squibb Co, Princeton, NJ 08648 USA; 9 Department of Surgery, Johns Hopkins University School of Medicine, Baltimore, MD 21287 USA; 10 Department of Gastrointestinal and Pancreatic Surgery, Zhejiang Provincial People’s Hospital, People’s Hospital of Hangzhou Medical College, Hangzhou, Zhejiang 310014 China; 11 Mays Cancer Center, University of Texas Health San Antonio MD Anderson, San Antonio, USA; 12 Division of BiliarySurgery, Department of General Surgery, West China Hospital, Sichuan University, Chengdu, Sichuan, 610041, China

**Keywords:** Pancreatic ductal adenocarcinoma, STING agonist, NLRP3 agonist, Innate immune agonists, Immune checkpoint inhibitors, Chemotherapy, Tumor microenvironment

## Abstract

**Background:**

The combination of conventional chemotherapy and immune checkpoint inhibitors (ICIs) has been unsuccessful for pancreatic ductal adenocarcinoma (PDAC). Administration of maximum tolerated dose of chemotherapy drugs may have immunosuppressive effects.

**Methods:**

We thus tested, by using the preclinical model of PDACs including the genetically engineered mouse KPC spontaneous pancreatic tumor model and the pancreatic KPC tumor orthotopic implant model, the combinations of synthetic innate immune agonists including STING and NLRP3 agonist, respectively, and ICIs with or without chemotherapy.

**Results:**

We found that innate agonists potentiate the role of chemotherapy in inducing effector T cells and subsequently to prime the tumor microenvironment (TME) better for ICI treatments. Triple combination of chemotherapy, innate agonists, and ICIs is superior to single modalities or double modalities in antitumor efficacies. Adding chemotherapy to innate agonists enhances the infiltration of overall CD8^+^ T cells and the memory cytotoxic subtype. NLRP3 agonist has a less effect than STING agonist on driving the T cell exhaustion. Adding chemotherapy to innate agonists enhances the infiltration of dendritic cells (DCs) in the tumors and CD86^+^ mature DCs in tumor draining lymph nodes. RNA sequencing analysis of the pancreatic tumors demonstrates the role of the combination of STING/NLRP3 agonist and chemotherapy, but not either treatment modality alone, in upregulating the T cell activation signaling. The NLRP3 agonist-mediated T cell activation is likely through regulating the nitrogen metabolism pathways.

**Conclusion:**

This study supports the clinical testing of both STING and NLRP3 agonists, respectively, in combination with chemotherapy to sensitize PDAC patients for ICI treatments.

**Supplementary Information:**

The online version contains supplementary material available at 10.1186/s40364-024-00721-7.

## Background

Pancreatic ductal adenocarcinoma (PDAC) is an aggressive malignant disease with a 5-year survival rate of approximately 12% [1]. Radiation and chemotherapy have demonstrated limited improvement in survival as the response duration is short. Immune checkpoint inhibitors (ICIs) induce a durable response in various tumor types [2]. However, PDAC is typically resistant to ICIs. ICI-resistant tumor types, also known as immunologically cold tumors, are characterized by ‘exclusion’ or desert of T lymphocytes [3, 4].

Previous preclinical and clinical studies have found that cancer vaccine therapy can convert PDAC from a ‘cold’ tumor into a ‘hot’ one [5–10]. Synthetic innate immune agonists are known to provide the priming mechanisms to convert cold tumors to hot ones [11]. Chemotherapy and radiation are traditional cancer therapies that may offer an in-situ vaccination effect by inducing immunogenic cell death (ICD) [12]. Strategies that successfully reprogram the TME in “cold” tumors such as PDAC may overcome their resistance to ICIs and ultimately lead to the development of rational combination therapies to extend the clinical benefits of ICIs to a broader range of cancer types and patient population.

The role of the innate immune system in cancer immunosurveillance and induction of antitumor immune responses has been well recognized [13]. Cells that can mediate innate immune response have pattern recognition receptors (PRRs) on their surfaces, including the retinoic acid-inducible gene-I (RIG-I)-like receptors (RLRs), the DNA-sensing cGAS/stimulator of interferon genes (STING) pathway, the nucleotide-binding oligomerization domain-like receptors (NLRs), and Toll-like receptors (TLRs). Pathogens or the components in the pathogens activate the PRRs and provide damage-associated molecular patterns (PAMP/DAMPs). Synthetic innate agonists that structurally resemble PAMPs but lack the infectious capacities of pathogens engage the innate immune cells by inducing inflammatory cytokines, chemokines, and co-stimulatory molecules and accompanying the maturation of dendritic cells (DCs) and trafficking of DCs to lymph nodes, where the innate immune response transits to the adaptive immunity and activates antigen-specific T cells. Several small molecule innate agonists have been developed as immunotherapeutics or vaccine adjuvants in various cancers to pursue their antitumor potential [14].

Synthetic cyclic dinucleotides (CDNs) are the first generation of STING agonists that entered the clinical trial phase of drug development due to their structural versatility and ability to bind all prevalent allelic variants of human STING. MK-1454, a synthetic CDN, was tested as monotherapy or in combination with anti-PD-1 antibody (α-PD-1) pembrolizumab for the treatment of advanced solid tumors (NCT03010176, NCT04220866) [15]. Another CDN, ADU-S100 (also known as MIW815), was tested in a clinical trial (NCT03937141) for patients with advanced/metastatic solid tumors or lymphomas and demonstrated its safety [16]. ADU-S100 was tested in combination with α-PD-1 spartalizumab (NCT03172936) [17] and anti-CTLA-4 antibody (α-CTLA-4) ipilimumab (NCT02675439) in patients with solid tumors or lymphomas, respectively. However, antitumor efficacies of STING agonists have yet to be substantiated, primarily due to the challenge of intratumoral delivery of such agents that has limited the testing of these agents for efficiency in appropriate disease indications such as PDAC.

Due to the challenge presented by intratumor delivery, STING agonists have yet to make it beyond preclinical studies to be tested in PDAC patients for their efficacy [18, 19]. BMS-986,301 is a next-generation, CDN-based STING agonist and has demonstrated more than 90% tumor regression compared to only 13% with ADU-S100 in the preclinical models [20]. Our prior study showed that systemic delivery of this STING agonist is superior to the intratumoral delivery in the mouse models of PDAC that resemble human diseases [21]. The phase I study of BMS-986,301 (NCT03956680) also demonstrated the safety of its systemic delivery.

NLR family pyrin domain containing 3 (NLRP3) belongs to the NLR subfamily of PRRs. The caspase-1 activating complex, also called the NLRP3 inflammasome, is formed by NLRP3 and the adaptor ASC protein PYCARD [22, 23]. The involvement of NLRP3 inflammasome in tumorigenesis is shown by multiple studies [24–27]. BMS-986,299 is an imidazole-fused 2-aminoquinoline NLRP3 agonist [28] and has demonstrated antitumor activities in the preclinical models. Our prior study also showed that systemic delivery of this NLRP3 agonist is equivalent to the intratumoral delivery in the mouse models of PDAC that resemble human diseases [21]. The unpublished phase I study of BMS-986,299 (NCT03444753) also demonstrated the safety of its systemic delivery.

The role of chemotherapy in treating PDAC is well-established for all stages of PDAC [29]. Several features of dying tumor cells serve as DAMPs to trigger an immune response [30]. Liposomal doxorubicin, which induces ICD, was effective in immunocompetent mice with implanted tumors but not in immunocompromised nude mice, highlighting the importance of adaptive immunity for the efficacy of this chemotherapy [31]. However, chemotherapy-induced ICD alone has not consistently induced an immune response that renders an antitumor activity sufficiently. On the other hand, common chemotherapy drugs, in addition to direct effects on cancer cells, can also influence immune cells, which however highly depends on the type, dose, and administration schedule of these drugs [32]. Administration of the maximum tolerated dose of chemotherapy drugs may have strong immunosuppressive effects, reducing the number of effector cells such as CD8^+^ T cells and NK cell. In contrast, administrating low doses of chemotherapy drugs may conversely potentiate immune response by decreasing immune inhibitory cells such as myeloid-derived suppressor cells (MDSCs) and T regulatory cells (Tregs) [33], however, may not be sufficient for an adequate antitumor cytotoxic activity.

The combination of conventional chemotherapy and ICIs has become a part of the standard of care for treating breast cancer, non-small‐cell lung cancer, and gastro‐esophageal carcinomas [34]. Nevertheless, this strategy has been unsuccessful for PDAC. Therefore, in this study, we hypothesize that synthetic innate agonists may synergize with chemotherapy-induced DAMP signaling to potentiate effector T cells and prime the TME better for the ICI treatments. As STING agonist and NLRP3 agonist have different mechanisms, we also compared the preclinical efficacy and underlying mechanisms of action between these two different synthetic innate agonists in combination with chemotherapy.

## Methods

### Cell lines

The KPC cell line is a previously established murine PDAC tumor cell line [35] that was derived from a KPC (*LSL-KrasG12D/+; LSL-Trp53R172H/+; Pdx-1-Cre*) mouse on a C57BL/6 background with *Kras* and *p53* mutations, whose pancreatic tissue-specific knock-in was manipulated by the *Cre* recombinase under the control of a PDX1 (pancreatic and duodenal homeobox 1) promoter. The KPC cell line was cultured and passaged following previously established procedures [35, 36].

### Mice and in vivo experiments

The in vivo study was conducted in accordance with the guidelines established by the Institutional Animal Care and Use Committee (IACUC) of the Johns Hopkins University.

#### KPC model

**Fig. 1:**
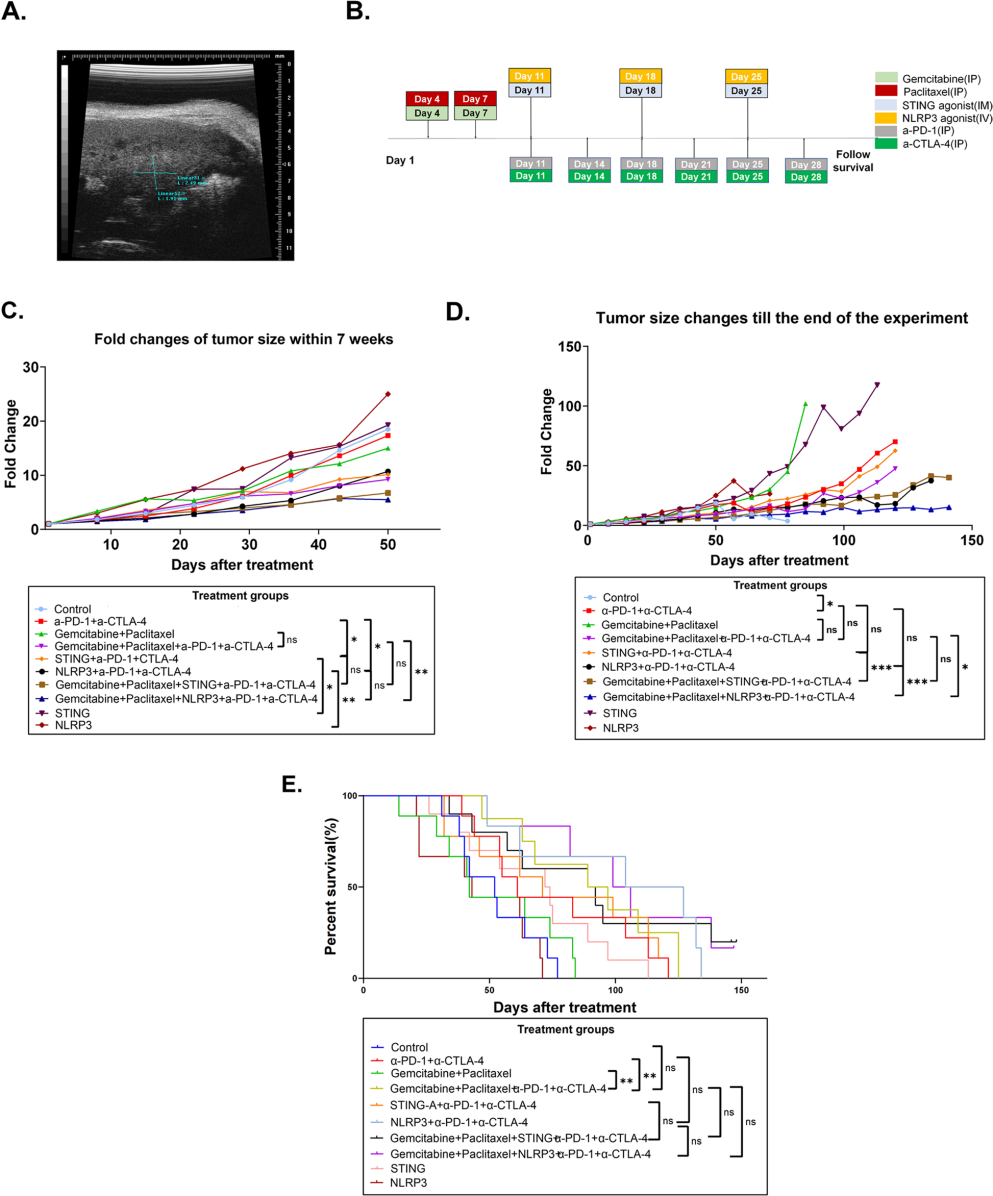
Antitumor activity of combination chemotherapy, innate immune agonists, and ICIs in the mouse spontaneous tumor model of pancreatic cancer. **A **Ultrasonographic measurement for the pancreatic lesion before treatment. **B **Treatment schema. KPC mice with spontaneously developed pancreatic tumors were treated with gemcitabine and paclitaxel via intraperitoneal (IP) injection twice a week for one week. Anti-PD-1 antibody and anti-CTLA-4 antibody were IP administered twice a week for three weeks. STING agonist and NLRP3 agonist were administered via intramuscular (IM) injection or intravenous (IV) injection, respectively, once a week for three weeks. **C** Tumor growth curves during the first 7 weeks were measured by ultrasound and compared among treatment groups as indicated by two-way Anova test. **D **Tumor growth curves till the end of the experiment were measured by ultrasound and compared among treatment groups as indicated by two-way Anova test. **E **Kaplan-Meier survival curves are compared among treatment groups by Log-rank test. Mice number involved in Control, α-PD-1 + α-CTLA-4, chemo, chemo > α-PD-1 + α-CTLA-4, STING/α-PD-1 + α-CTLA-4, NLRP3/α-PD-1 + α-CTLA-4, chemo > STING/α-PD-1 + α-CTLA-4, chemo > NLRP3/α-PD-1 + α-CTLA-4, STING, NLRP3 was 9, 9, 9, 8, 9, 6, 10, 6, 10, 9, respectively. *p < 0.05; **p < 0.01; ***p < 0.001. NS, not significant

The KPC mouse model of PDAC was first described in 2005 [37]. This mouse model spontaneously develops PDAC. KPC mice were subjected to weekly to twice weekly ultrasonic screening at age of 3 months and enrolled in the experiment once either the length, width, or height of the pancreatic tumor reached 2 mm (Fig. 1A) to ensure eligible mice had equivalent tumor burdens. Consecutive, eligible KPC mice were enrolled in the experiment in Fig. 1B and randomized to the ten treatment groups over a period of 8 months to ensure unbiasedness and reproducibility.

#### Orthotopic model

The pancreatic tumor orthotopic implant model using KPC cells was established following previously published methods [35]. Briefly, 2 × 10^6^ KPC cells were subcutaneously injected into the flanks of syngeneic female C57BL/6 mice. After 1 to 2 weeks, the subcutaneous tumors were harvested and dissected into 2–3 mm^3^ cubes. New syngeneic female C57BL/6 mice at ages of 8 to 10 weeks were anesthetized; and a left subcostal incision was made in the abdomen to expose the body and tail of the pancreas. A small pocket was created in the middle of the pancreas using micro-scissor; and a single 2–3 mm^3^ piece of the subcutaneous tumor was implanted into the small pocket. The incision in the pancreas was closed using 7 − 0 Prolene suture and the abdominal wall was sutured using 4 − 0 sutures. The implantation of a tumor cube instead of the injection of tumor cells allows the monitoring of a discrete tumor by ultrasound.

#### Tumor treatment

For the KPC mouse model, once the eligible tumor was confirmed by small-animal ultrasound (Vevo770, VisualSonics), the following treatments were initiated. For the orthotopic model, 3 days after tumor implantation (Day 4), the following treatments were initiated. Both gemcitabine(50 mg/kg) and paclitaxel (10 mg/kg) were intraperitoneally injected twice a week for one week. STING agonist (100 mg) and NLRP3 agonist (100 mg) were given via intramuscular injection or intravenous injection once a week, respectively, for three consecutive weeks. Anti-mouse PD-1 antibodies (10 mg/kg; BMS) or isotype control IgG1 (10 mg/kg; BMS) were administered intraperitoneally twice weekly for a total of six doses. Anti-mouse CTLA-4 antibodies (10 mg/kg; BMS) or isotype control IgG2a (10 mg/kg; BMS) were administered intraperitoneally twice weekly for a total of six doses as well. Treatment toxicity was assessed via monitoring the mouse body weight and specific signs including bleeding, infection, paralysis, etc. The mice of both the KPC mouse model and the orthotopic model were subjected to daily monitoring. Tumor size was assessed on a weekly or twice weekly basis using small-animal ultrasound (Vevo770, VisualSonics). Mice displaying signs of distress such as hunched posture and lethargy were considered to have reached survival endpoint and were subsequently euthanized according to the IACUC-approved protocol.

### Cell staining and flow cytometry

On Day 15 in the above-described experiment with the orthotopic model, half of the mice in each treatment group were euthanized; and the pancreatic tumors were collected for immune cell analysis. The tumor samples were mechanically minced using the gentleMACS Dissociator with the Tumor Dissociation Kit specifically designed for tumor samples (Miltenyi Biotec, San Diego, CA, USA); and tumor infiltrating leukocytes were harvested as previously described [38]. Next, the leukocytes isolated from tumor samples were stained using the Live Dead Aqua Dead Cell Kit (Invitrogen) to distinguish live and dead cells. Following that, mouse Fc antibody (BD Pharmingen) was used to block nonspecific binding by incubating the leukocytes with it for 10 mins on ice. Subsequently, cell surface marker antibodies (Table S1) were added to stain the cells for a 30-minute incubation on ice. The cells were then washed twice and resuspended in the FACS buffer. Flow cytometry analysis was performed using the CytoFLEX (Beckman Coulter); and the resulting data were analyzed using CytExpert software (Beckman Coulter).

### RNA sequencing

TRIzol Reagent (Thermo Fisher Scientific) was utilized to extract total RNA from the tumor-infiltrating leukocyte pellets isolated from the tumors. The extracted RNA was then subjected to the RNA sequencing, which was performed at BGI Genomics. Gene enrichment analysis was performed by the KEGG method.

### Statistical analyses

Statistical analysis was performed using the GraphPad Prism software. Data were presented as the mean ± SEM (standard error of the mean). The number of samples used in the analysis was specified in the corresponding figure legends. Kaplan-Meier curves and log-rank tests were applied to estimate the median survival and to compare survival rates between treatment groups. Tumor growth curves were compared using the ANOVA test method. A p-value less than 0.05 was considered statistically significant.

## Results

### Antitumor activity of the combination therapy with chemotherapy, innate agonists, and immune checkpoint inhibitors in the mouse spontaneous tumor model of pancreatic cancer

Previously, our preclinical study has established the intramuscular injection for the STING agonist and the intravenous injection for the NLRP3 agonist as their systemic administration routes to be tested in the clinical trials [21]. In that preclinical study, we demonstrated that the STING agonist treatment in a dual ICIs combination with α-PD-1 and α-CTLA-4, comparing to STING agonist alone or ICI antibodies alone, significantly prolonged the survival of the KPC mouse model with conditional knock-in of Kras and p53 mutations. However, the survival benefit of the STING agonist in combination with the dual ICIs combination was still modest. Thus, in this study, we examined the synergistic effect of the standard of care chemotherapy and STING agonist or NLRP3 agonist. KPC mice (3–4 months of age) were randomized into ten treatment groups. Weekly ultrasounds were performed to identify tumors with a size of 1–3 mm in diameter (Fig. 1A). Treatment groups and schemas were described in Fig. 1B and Table S2. Gemcitabine, paclitaxel or PBS was administered intraperitoneally twice a week for a total of one week followed by STING agonist, NLRP3 agonist, or their respective vehicle control was administrated intramuscularly once a week, NLRP3 agonist was administrated intravenously once a week for a total of three weeks. α-PD-1, α-CTLA-4, or their respective isotype control antibody was administrated intraperitoneally twice weekly for three weeks.

Tumor volumes were measured once or twice weekly by ultrasound until the mice reached survival endpoint (Fig. 1C and D; Figure S1). We first compared the tumor sizes among treatment groups on Day 49 before significant numbers of deaths in the mice occurred (Fig. 1C). The comparison results were consistent with our prior results [21], showing that tumor size on Day 49 in the STING/α-PD-1 + α-CTLA-4 group was significantly smaller than that in the STING agonist alone or the ICIs alone group (*p* = 0.018 and 0.032, respectively). Similarly, tumor size on Day 49 in the NLRP3/α-PD-1 + α-CTLA-4 group was significantly smaller compared to the NLRP3 agonist alone or the ICIs alone group (*p* = 0.009 and 0.029, respectively). Interestingly, adding gemcitabine and paclitaxel to ICIs (the Chemo > α-PD-1 + α-CTLA-4 treatment group) resulted in a similar antitumor activity comparing to STING/α-PD-1 + α-CTLA-4 or NLRP3/α-PD-1 + α-CTLA-4. A modest activity of the Chemo > α-PD-1 + α-CTLA-4 treatment was observed in the KPC mice previously [21] although the mice responded heterogeneously to this treatment combination. Nevertheless, both the chemo > STING/α-PD-1 + α-CTLA-4 and the chemo > NLRP3/α-PD-1 + α-CTLA-4 groups showed slower tumor growth than the chemo > α-PD-1 + α-CTLA-4 group, respectively, especially the NLRP3 agonist-based combination therapy, which reached statistical significance **(**Fig. 1C). We also observed that approximately 90% of mice died from a large, dominant primary pancreatic tumor and less than 10% of mice died from extensive metastases post 49 days of initial treatment. After Day 49, most mice (approximately 80%) were found to have both a large primary tumor and at least one metastatic focus. With these findings, it would be difficult to determine the main cause of death. However, STING/α-PD-1 + α-CTLA-4 and NLRP3/α-PD-1 + α-CTLA-4 treatment groups exhibited extensive metastases which may have contributed to their deaths. Likely due to the variation in metastasis burden among mice in different treatment groups, the survival of mice in the Chemo > STING/α-PD-1 + α-CTLA-4 and Chemo > NLRP3/α-PD-1 + α-CTLA-4 treatment groups was not significantly improved compared to those in the chemo > α-PD-1 + α-CTLA-4 group (*p* = 0.633 and 0.316, respectively) (Fig. 1E; Figure S2). Nevertheless, only mice that survived at the end of the experiment (Week 20) were from the Chemo > STING/α-PD-1 + α-CTLA-4 and Chemo > NLRP3/α-PD-1 + α-CTLA-4 treatment groups. In addition, compared to control, α-PD-1 + α-CTLA-4, chemo > α-PD-1 + α-CTLA-4, STING agonist alone, STING/α-PD-1 + α-CTLA-4, NLRP3/α-PD-1 + α-CTLA-4, Chemo > STING/α-PD-1 + α-CTLA-4, and Chemo > NLRP3/α-PD-1 + α-CTLA-4 all demonstrated a meaningful improvement of survival in KPC mice. However, the chemotherapy effect appeared to be minimal. Although it was of interest to observe the antitumor activity of α-PD-1 + α-CTLA-4 in KPC mice, the survival prolongation by α-PD-1 + α-CTLA-4 was not significant. Compared to α-PD-1 + α-CTLA-4 or STING agonist alone, we did not observe survival improvement by combining STING agonist with ICIs (*p* = 0.795 and 0.266, respectively), likely due to the heterogeneity of KPC mice in developing metastasis. On Day 49, the mice with extensive metastases were all from the treatment groups with STING/α-PD-1 + α-CTLA-4. However, compared to ICIs, we observe a near significant improvement of survival by combining NLRP3 agonist with ICIs (*p* = 0.055) (Fig. 1E, Figure S2). We noticed that survival could be affected by both primary tumor growth and metastasis. For example, mice in the control group died from metastases when the primary pancreatic tumors were still small. It is possible that different treatment combinations may affect primary tumors and metastases differently. This may explain the lack of statistical significance in the comparisons of survival between some groups that showed significant differences in the primary tumor growth. We did not observe any obvious sign of toxicity including bleeding, infection, paralysis, and weight loss as a result of treatment, suggesting chemotherapy in synergy with innate immune agonists was safe in treating preclinical tumors of PDAC. In summary, combining primary tumor growth assessment and survival assessment, this experiment with the KPC mouse model that develops PDAC spontaneously by resembling clinical trials in human PDAC patients demonstrated that the antitumor activity of the triple combination with chemotherapy, innate immune agonists, and ICIs is likely superior to single modalities, particularly the standard of care chemotherapy, and double modalities although the indispensable role of each component in this triple modality combination would need to be further verified by human patient studies.

### Antitumor activity of the combination therapy with chemotherapy, innate agonists, and immune checkpoint inhibitors in the mouse orthotopic transplant tumor model of pancreatic cancer

**Fig. 2:**
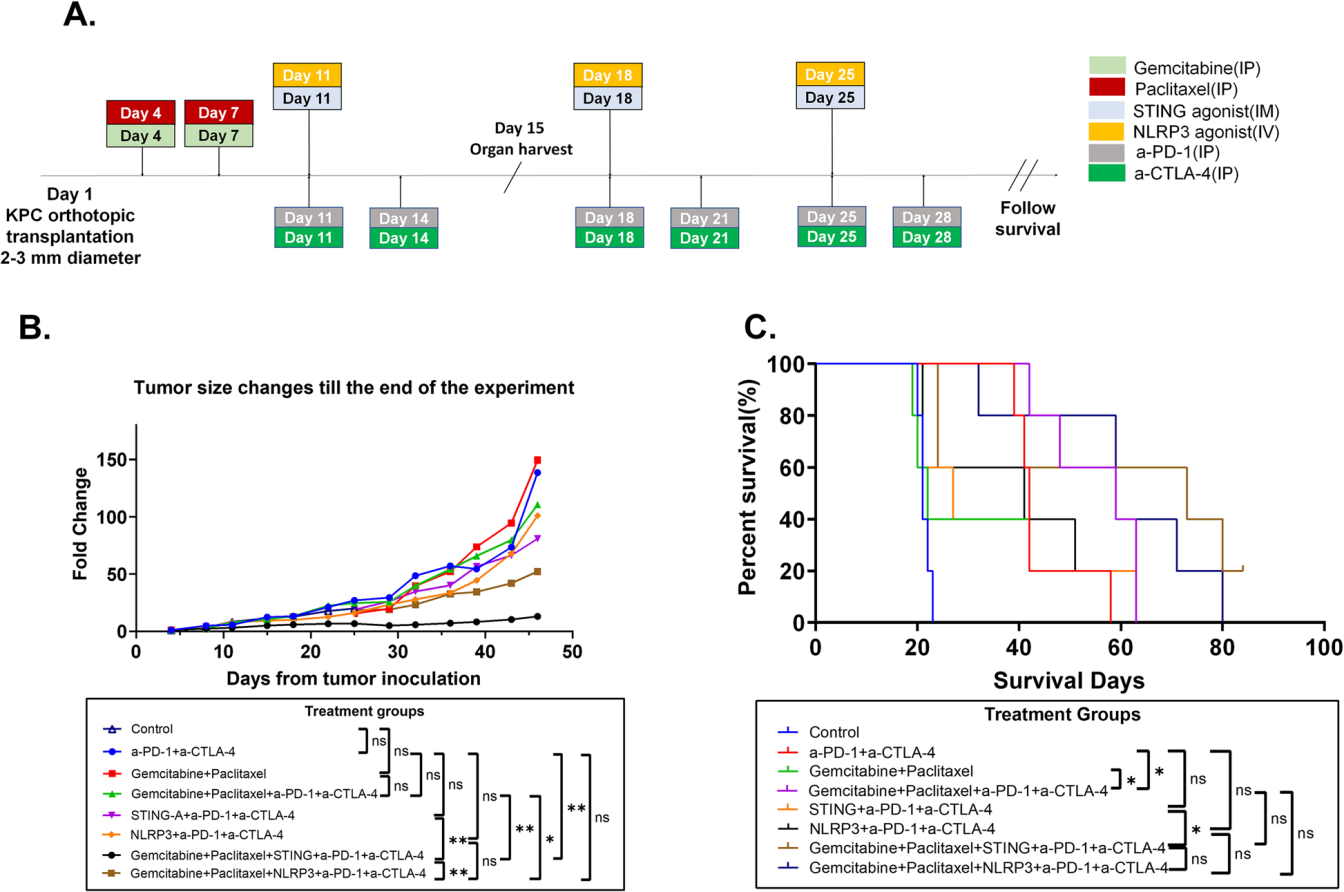
Antitumor activity of combination chemotherapy, innate immune agonists, and ICIs in the mouse orthotopic transplant tumor model of pancreatic cancer. **A** Treatment schema similarly as described in **A**. **B** Tumor growth curves during the first 7 weeks were measured by ultrasound and compared among treatment groups as indicated by two-way Anova test (*n*=5 per group). **C** Kaplan-Meier survival curves are compared among treatment groups by Log-rank test (*n*=5 per group). *p<0.05; **p<0.01. NS, not significant

To further investigate the effectiveness of triple modality combinations, specifically the Chemo > STING/α-PD-1 + α-CTLA-4 and Chemo > NLRP3/α-PD-1 + α-CTLA-4 treatments, we examined 8 out of the above 10 treatments in the mouse model with orthotopically implanted KPC tumors. These tumors were considered more homogeneous compared to spontaneously formed KPC tumors. Three days after tumor implantation, mice were randomly assigned to 8 different treatment groups (Fig. 2A; Figure S3; Figure S4; Table S3) for evaluation.

Primary pancreatic tumors were measured once to twice weekly by ultrasound until mice reached the survival endpoint (Fig. 2A; Figure S3; Figure S4). Adding STING agonist or NLRP3 agonist to the chemo > α-PD-1 + α-CTLA-4 treatment led to a significantly enhanced antitumor activity compared to the chemo > α-PD-1 + α-CTLA-4 treatment (Fig. 2B; Figure S3). The chemo > STING/α-PD-1 + α-CTLA-4 treatment had significantly better antitumor activity than STING/α-PD-1 + α-CTLA-4 (*p* = 0.005) while chemo > NLRP3/α-PD-1 + α-CTLA-4 had a statistically non-significant improvement on the antitumor activity comparing to NLRP3/α-PD-1 + α-CTLA-4 (*p* = 0.466) (Fig. 2B). Consistently, mice treated with chemo > STING/α-PD-1 + α-CTLA-4 had significantly better survival than STING/α-PD-1 + α-CTLA-4 (*p* = 0.034) while mice treated with chemo > NLRP3/α-PD-1 + α-CTLA-4 had better survival, which was nearly significant (*p* = 0.055), than NLRP3/α-PD-1 + α-CTLA-4 (Fig. 2C). Interestingly, the results showed that the chemo > STING/α-PD-1 + α-CTLA-4 treatment had significantly better tumor growth suppression than chemo > NLRP3/α-PD-1 + α-CTLA-4 (Fig. 2B). Nevertheless, mice treated with chemo > STING/α-PD-1 + α-CTLA-4 exhibited similar survival to those treated with chemo > NLRP3/α-PD-1 + α-CTLA-4(*p* = 0.464) (Fig. 2C; Figure S4). Taken together, these results support the combination of a STING or NLRP3-based immunotherapy strategy with chemotherapy.

### Adding chemotherapy to innate agonists enhances the infiltration of effector T cells in general and memory cytotoxic T cell subtype

**Fig. 3:**
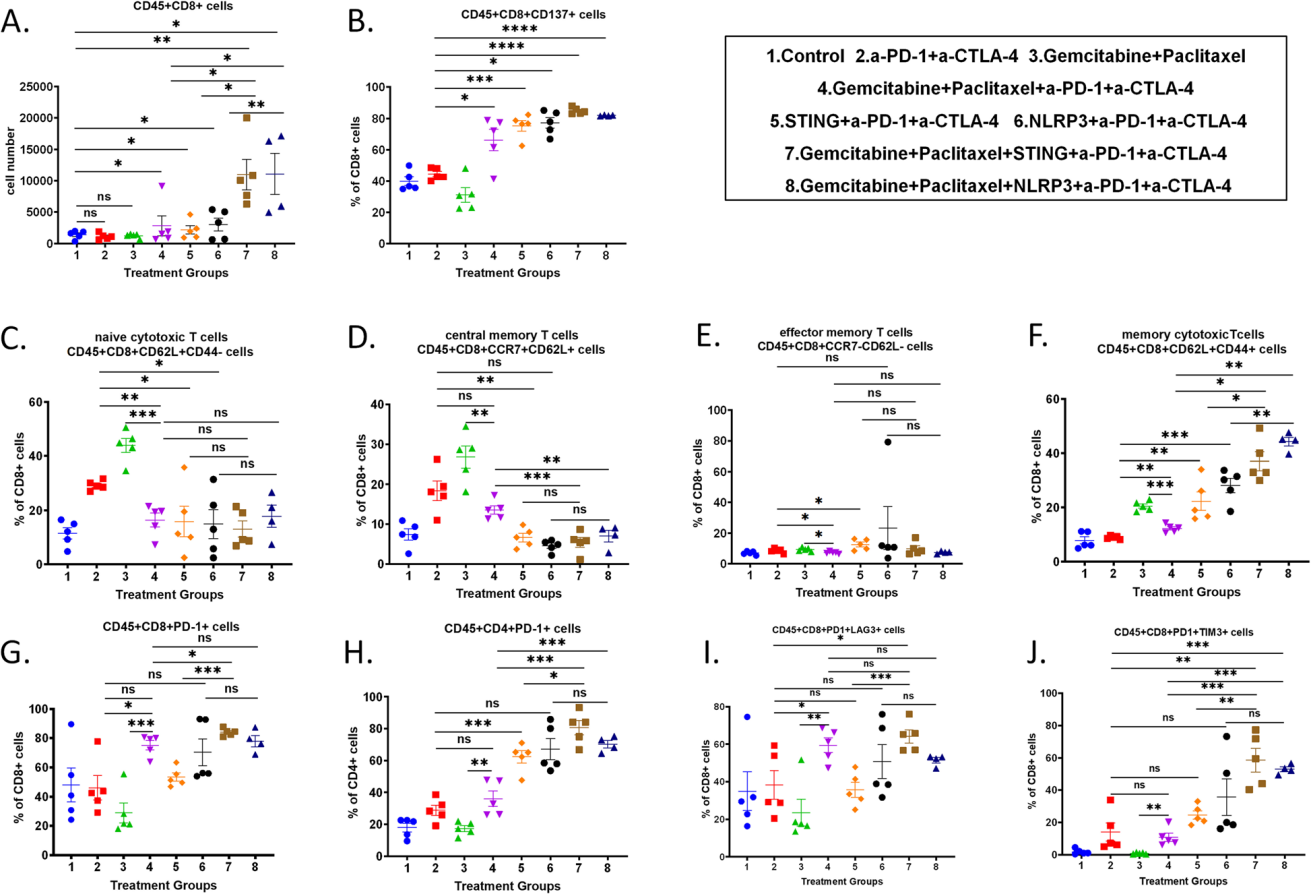
The effects of STING or NLRP3 agonists and their combinations on the infiltration of effector T cells in general and memory cytotoxic T cell subtype in orthotopically implanted tumor model. **A** The number of CD45^+^CD8^+^ T cells in the tumors per gram of tumor. **B** Percentages of CD8^+^CD137^+^ T cells among CD8^+^ T cells in the tumors. **C**-**G** Percentages of CD62^+^CD44^-^ naïve cytotoxic T cells (**C**), CCR7^+^CD62L^+^ central memory T cells (**D**), CCR7^-^CD62L^-^ effector memory T cells (**E**), CD62L^+^CD44^+^ memory cytotoxic T cells (**F**), and PD-1^+^CD8^+^ T cells (**G**) among CD8^+^ T cells in the tumors. **H** Percentages of PD-1^+^CD4^+^ T cells among CD4^+^ T cells in the tumors. **I** Percentages of PD-1^+^LAG3^+^CD8^+^ T cells among CD8^+^ T cells in the tumors. **J** Percentages of PD-1^+^TIM3^+^CD8^+^ T cells among CD8^+^ T cells in the tumors (*n*=5 per group except *n*=4 in chemo>NLRP3/α-PD-1+α-CTLA-4). The experiment was repeated two to three times. Data shown as mean ± SEM and compared by unpaired t test; *p<0.05; **p<0.01; ***p<0.001; ****p<0.0001. NS, not significant

To understand how the combination of chemotherapy, innate agonists, and ICIs modulates the antitumor activity, we analyzed the tumor infiltrating leukocytes (TILs) by flow cytometry (Figure S5) from the orthotopically implanted tumors following a short course of treatment before the tumor size was significantly affected by the treatment effect. Mice with orthotopically implanted tumors were randomized into 5 mice per group to receive the same dose and schedule of treatments as described above except for euthanizing occurred on Day 15 (Fig. 2A). Compared to the control group, the absolute numbers of CD45^+^CD8^+^ cells in the tumor, normalized by tumor weight, were significantly higher in the chemo > STING/α-PD-1 + α-CTLA-4 and chemo > NLRP3/α-PD-1 + α-CTLA-4 groups, with an increase exceeding 10-fold. In contrast, no significant increase was observed in the α-PD-1 + α-CTLA-4 or chemo groups. Furthermore, when compared to the chemo > α-PD-1 + α-CTLA-4 group, the number of CD45^+^CD8^+^ cells was significantly higher in both the chemo > STING/α-PD-1 + α-CTLA-4 and chemo > NLRP3/α-PD-1 + α-CTLA-4 groups (Fig. 3A). These results suggested that combining chemotherapy and innate agonist has a synergistic effect on inducing CD8^+^ T cell infiltration into PDAC tumors. The patterns of CD8^+^ T cell subtypes including CD45^+^CD8^+^CD137^+^ cells, CD45^+^CD8^+^OX40^+^ cells, and CD45^+^CD8^+^PD-1^+^ cells are essentially the same as that of CD8^+^ T cells (Figure S6A-C). To examine the treatment effect on the T cell activation pathway, the percentage of CD137^+^ T cells or OX40^+^ T cells among CD8^+^ T cells was examined. We chose to examine the percentages of CD8^+^ T cell subtypes instead of the absolute numbers of CD8^+^ T cell subtypes per tumor weight because we were interested in understanding how the treatments changed the function of CD8^+^ T cell subtypes within tumor-infiltrating CD8^+^ T cells. Nevertheless, the percentages of CD8^+^ T cell subtypes were largely consistent with the absolute numbers of CD8^+^ T cell subtypes per tumor weight (Figure S7). Compared with the control group, the percentage of CD137^+^ T cells among CD8^+^ cells was increased in all treatment groups except the chemo group. Compared with the α-PD-1 + α-CTLA-4 group, the percentage of CD137^+^ T cells among CD8^+^ cells was significantly increased in the chemo > α-PD-1 + α-CTLA-4, STING/α-PD-1 + α-CTLA-4, NLRP3/α-PD-1 + α-CTLA-4, chemo > STING/α-PD-1 + α-CTLA-4, or chemo > NLRP3/α-PD-1 + α-CTLA-4 group, suggesting that both chemotherapy and innate agonists promote the T cell activation (Fig. 3B) and chemotherapy itself may also serve as an innate agonist. In contrast, compared with the α-PD-1 + α-CTLA-4 group, the percentage of CD137^+^ T cells among CD4^+^ cells was significantly increased in the STING/α-PD-1 + α-CTLA-4, NLRP3/α-PD-1 + α-CTLA-4, chemo > STING/α-PD-1 + α-CTLA-4, or chemo > NLRP3/α-PD-1 + α-CTLA-4 group, but not the chemo > α-PD-1 + α-CTLA-4 group (Figure S6D). Combining innate agonists with ICIs does not appear to have an impact on the OX40-mediated T cell activation (Figure S6E-F).

We also examined the cytotoxic and memory T cell compositions within CD8^+^ T cells population. Both percentages of naïve T cells (Fig. 3C) and central memory T cells (Fig. 3D) among CD8^+^ T cells were significantly increased in the chemo or α-PD-1 + α-CTLA-4 group when compared to the control group. However, the combination of chemo or innate agonist with α-PD-1 + α-CTLA-4 led to a substantial decrease of these forementioned T cell subtypes. The percentage of effector memory T cells remained low in all treatment groups (Fig. 3E). Interestingly, adding either of innate agonists to α-PD-1 + α-CTLA-4 significantly increased the percentage of memory cytotoxic T cells compared to α-PD-1 + α-CTLA-4 (Fig. 3F). Adding the chemotherapy to STING/α-PD-1 + α-CTLA-4 or NLRP3/α-PD-1 + α-CTLA-4 significantly further increased the percentage of memory cytotoxic T cells (Fig. 3F).

### NLRP3 agonist is less efficacious than STING agonist in driving the T cell exhaustion

Compared to the α-PD-1 + α-CTLA-4 treatment, the percentage of PD-1^+^CD8^+^ T cells among CD45^+^CD8^+^ cells was significantly increased in their combination with chemo or in their combination with both chemo and innate agonists including Chemo > α-PD-1 + α-CTLA-4, chemo > STING/α-PD-1 + α-CTLA-4, and chemo > NLRP3/α-PD-1 + α-CTLA-4 treatments (Fig. 3G). The percentage of PD-1^+^ T cells among CD45^+^CD8^+^ cells was significantly higher in the chemo > STING/α-PD-1 + α-CTLA-4 group than the Chemo > α-PD-1 + α-CTLA-4 or STING/α-PD-1 + α-CTLA-4 group. Compared with the chemo > NLRP3/α-PD-1 + α-CTLA-4 treatment, the percentage of PD-1^+^ T cells among CD45^+^CD8^+^ cells in the chemo > STING/α-PD-1 + α-CTLA-4 group was significantly higher. The percentage of PD-1^+^CD4^+^ T cells among CD45^+^CD4^+^ cells showed similar trends in most of the treatment groups (Fig. 3H). These data may suggest that combining innate agonists with chemotherapy can induce CD8^+^PD-1^+^ and CD4^+^PD-1^+^ T cells. In contrast, compared to the α-PD-1 + α-CTLA-4 group, the percentage of PD-1^+^LAG3^+^ cells among CD8^+^ T cells was increased in chemo > α-PD-1 + α-CTLA-4, STING/α-PD-1 + α-CTLA-4, NLRP3/α-PD-1 + α-CTLA-4, chemo > STING/α-PD-1 + α-CTLA-4 and chemo > NLRP3/α-PD-1 + α-CTLA-4 groups; However, the increase was only statistically significant in the chemo > α-PD-1 + α-CTLA-4 and chemo > STING/α-PD-1 + α-CTLA-4 groups (Fig. 3I). Also compared to the α-PD-1 + α-CTLA-4 group, the percentage of PD-1^+^TIM3^+^ cells among CD8^+^ T cells was increased in the NLRP3/α-PD-1 + α-CTLA-4, chemo > STING/α-PD-1 + α-CTLA-4 and chemo > NLRP3/α-PD-1 + α-CTLA-4 groups; However, the increase was only statistically significant in the chemo > STING/α-PD-1 + α-CTLA-4 and chemo > NLRP3/α-PD-1 + α-CTLA-4 groups (Fig. 3J). These results suggest that NLRP3 agonist, compared to STING agonist, has a less effect on driving the T cell exhaustion.

### Adding chemotherapy to innate agonists enhances the infiltration of CD86 + mature DC cells in tumor draining lymph nodes

**Fig. 4:**
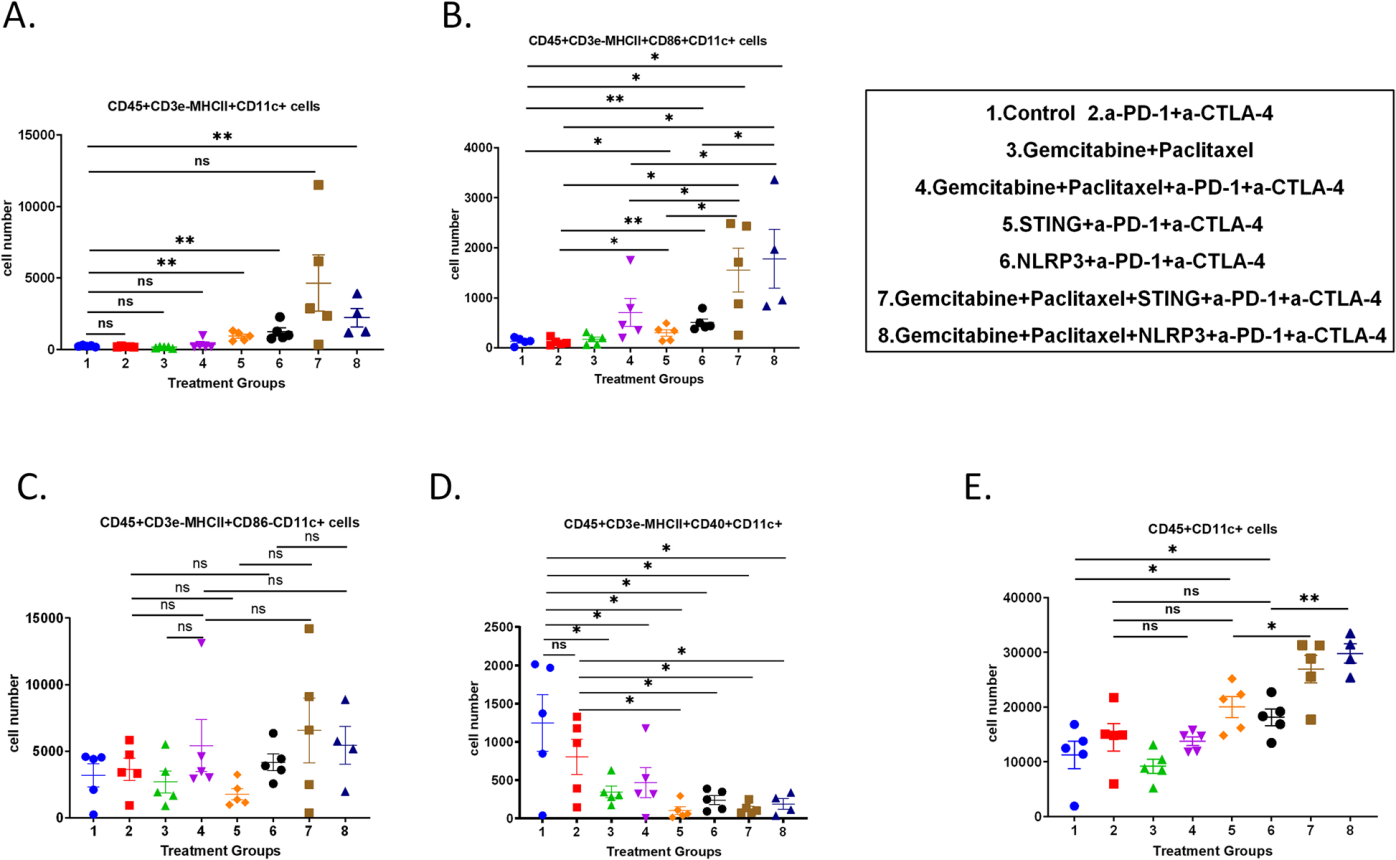
The effects of STING or NLRP3 agonists and their combinations on the antigen presenting process in the tumor draining lymph nodes from the orthotopically implanted KPC tumor mouse model. **A**-**E** The number of DCs (**A**), CD86^+^ DCs (**B**), CD86^-^DCs (**C**), CD40^+^ DCs (**D**), and CD45^+^CD11c^+^myeloid cells (**E**) in the draining lymph nodes per gram (n=5 per group except *n*=4 in chemo>NLRP3/α-PD-1+α-CTLA-4). The experiment was repeated two to three times. Data shown as mean ± SEM; compared by unpaired t test; *p<0.05; **p<0.01; NS, not significant

Next, we examined the effects of STING or NLRP3 agonists and their based combinations on the antigen presenting process in the orthotopically implanted KPC tumor model. Tumor-draining lymph nodes were harvested and digested into single-cell suspensions for flow cytometry analysis. Compared to the control group, the number of CD45^+^CD3e^−^MHCII^+^CD11c^+^ DCs in the tumor-draining lymph nodes was increased in all treatment groups except the α-PD-1 + α-CTLA-4 and chemo groups; and the difference was statistically significant in the STING/α-PD-1 + α-CTLA-4, NLRP3/α-PD-1 + α-CTLA-4, and chemo > NLRP3/α-PD-1 + α-CTLA-4 groups, but not in the chemo > STING/α-PD-1 + α-CTLA-4 group (Fig. 4A). However, the result of the chemo > STING/α-PD-1 + α-CTLA-4 group may be influenced by one mouse that had an extremely low number of DCs in the tumor-draining lymph nodes (Fig. 4A). This result suggested that innate agonists with or without chemotherapy are able to induce DCs in the tumor- draining lymph nodes.

We also examined the mature DC subtype, the CD45^+^CD3e^−^MHCII^+^CD86^+^CD11c^+^ cells in the tumor draining lymph nodes. Similar to the general DCs, compared to the control group or the α-PD-1 + α-CTLA-4 treatment group, the number of mature DCs increased in all treatment groups; and the difference was statistically different in the STING/α-PD-1 + α-CTLA-4, NLRP3/α-PD-1 + α-CTLA-4, chemo > STING/α-PD-1 + α-CTLA-4 or chemo > NLRP3/α-PD-1 + α-CTLA-4 treatment group (Fig. 4B). Compared to the chemo > α-PD-1 + α-CTLA-4 treatment group, the number of mature DCs was significantly increased in the chemo > STING/α-PD-1 + α-CTLA-4 or chemo > NLRP3/α-PD-1 + α-CTLA-4 group (Fig. 4B). On the other hand, compared to the STING/α-PD-1 + α-CTLA-4 group, the number of mature DCs increased in the chemo > STING/α-PD-1 + α-CTLA-4 group; and compared to the NLRP3/α-PD-1 + α-CTLA-4 treatment group, the number of mature DCs was significantly increased in the chemo > NLRP3/α-PD-1 + α-CTLA-4 group (Fig. 4B). In contrast, these differences were not seen with the CD86^−^ DCs (CD45^+^CD3e^−^MHCII^+^CD86^−^CD11c^+^ cells) (Fig. 4C). This result further suggested that innate agonists can induce mature DCs in the tumor draining lymph nodes in synergy with the chemotherapy treatment.

### Adding chemotherapy to innate agonists does not enhance the infiltration of CD40^+^DC cells in tumor draining lymph nodes

Compared to the control group, the cell number of the CD40^+^ DCs (CD45^+^CD3e^−^MHCII^+^CD40^+^CD11c^+^) in the tumor draining lymph nodes was significantly reduced in all treatment groups except α-PD-1 + α-CTLA-4 group (Fig. 4D). Compared with α-PD-1 + α-CTLA-4 group, the number of CD40^+^ DCs was significantly reduced in the STING/α-PD-1 + α-CTLA-4, NLRP3/α-PD-1 + α-CTLA-4, chemo > STING/α-PD-1 + α-CTLA-4, and chemo > NLRP3/α-PD-1 + α-CTLA-4 groups (Fig. 4D). Nevertheless, the percentages of CD40^+^ DCs among general DCs were low across all the control and treatment groups (Figure S8A). Compared to the α-PD-1 + α-CTLA-4 group, the percentage of CD86^+^ DCs among general DCs was significantly increased in the Chemo > α-PD-1 + α-CTLA-4, STING/α-PD-1 + α-CTLA-4, NLRP3/α-PD-1 + α-CTLA-4, chemo > STING/α-PD-1 + α-CTLA-4 or chemo > NLRP3/α-PD-1 + α-CTLA-4 group (Figure S8B). Compared to the STING/α-PD-1 + α-CTLA-4 or NLRP3/α-PD-1 + α-CTLA-4 group, the percentage of CD86^+^ DCs among general DCs was significantly further increased in the chemo > STING/α-PD-1 + α-CTLA-4 or chemo > NLRP3/α-PD-1 + α-CTLA-4 group, respectively (Figure S8B). These results suggest that chemotherapy-induced innate immune response, STING agonist, or NLRP3 agonist mainly target CD86^+^ DCs, but not CD40^+^ DCs.

### Adding chemotherapy to innate agonists enhances the infiltration of DC cells in the tumors

The total number of CD45^+^CD11c^+^ myeloid cells was significantly increased in the STING/α-PD-1 + α-CTLA-4, NLRP3/α-PD-1 + α-CTLA-4, chemo > STING/α-PD-1 + α-CTLA-4, and chemo > NLRP3/α-PD-1 + α-CTLA-4 groups, respectively (Figure S8C). CD45^+^CD11c^+^ myeloid cells also demonstrated a similar trend as the more defined DC population (CD45 + CD3e^−^MHCII^+^CD11c^+^ cells). Due to the marker limitation with the flow cytometry, we examined CD45^+^CD11c^+^ myeloid cells in the tumor as a surrogate of the DC population. The results demonstrated that the total number of CD45^+^CD11c^+^ myeloid cells were significantly increased in the STING/α-PD-1 + α-CTLA-4 or NLRP3/α-PD-1 + α-CTLA-4 group. However, it was not significantly increased in comparison to the α-PD-1 + α-CTLA-4 group (Fig. 4E). This result suggested that STING or NLRP3 agonist may not be sufficient in inducing antigen-presenting process in tumors. Combining chemo to α-PD-1 + α-CTLA-4 did not induce CD45^+^CD11c^+^ myeloid cells, either (Fig. 4E). Nevertheless, adding chemo to STING/α-PD-1 + α-CTLA-4 or NLRP3/α-PD-1 + α-CTLA-4 treatment significantly induced the CD45^+^CD11c^+^ myeloid cells in the tumors (Fig. 4E). It is possible that chemotherapy-induced tumor cell death released the tumor antigens, which synergized with STING and NLRP3 agonist-induced innate immune responses. These results further support the synergistic effect of combining chemo and innate agonists.

### RNA sequencing analysis demonstrates the role of the combination of innate agonists and chemotherapy in upregulating the T cell activation signaling and nitrogen metabolism pathways

**Fig. 5:**
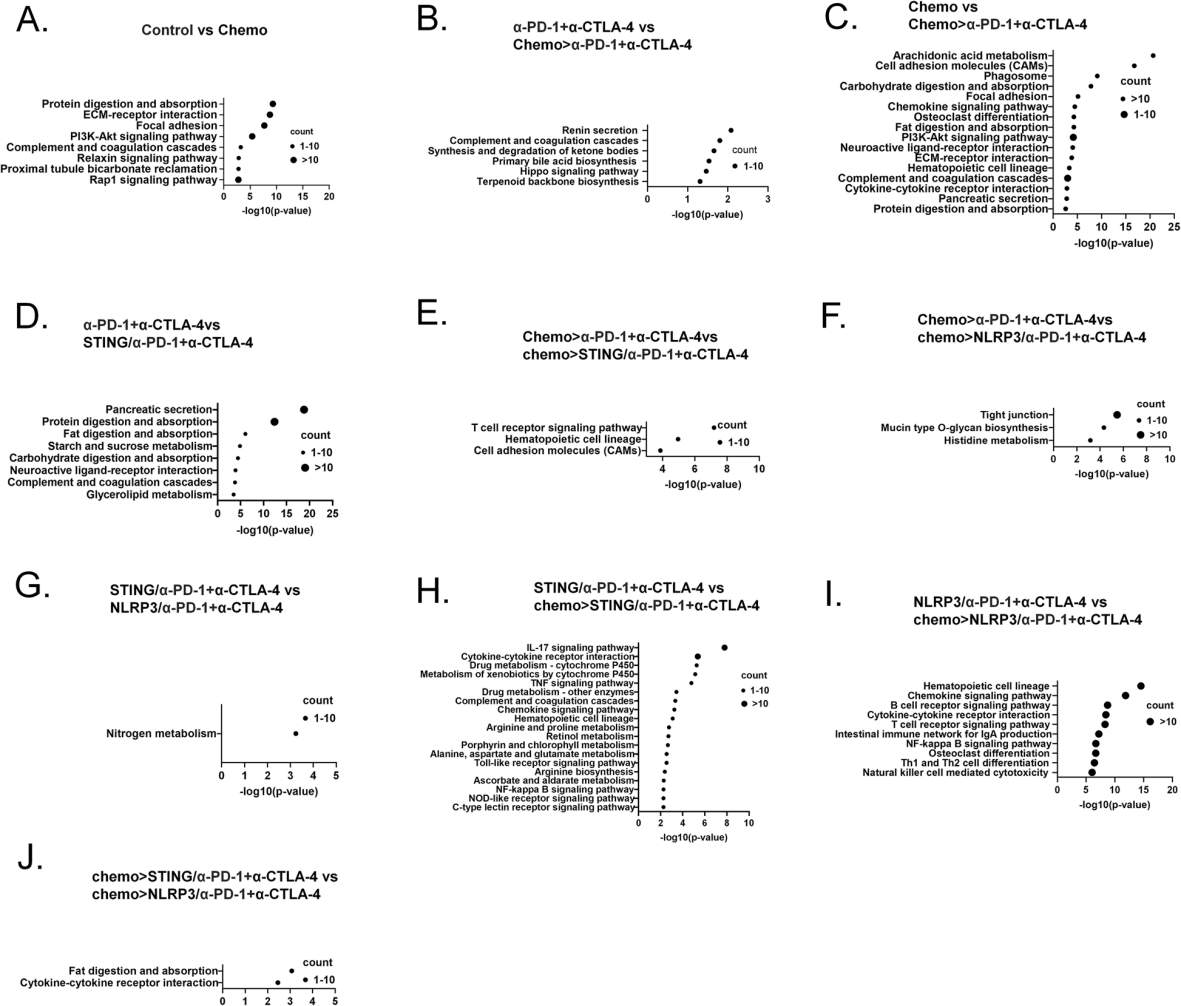
Gene enrichment analysis of orthotopically implanted tumors was carried out using KEGG methods. **A**-**I** Bubble plots of the differentially expressed genes comparing control vs chemo groups (**A**), α-PD-1+α-CTLA-4 vs chemo>α-PD-1+α-CTLA-4 groups (**B**), chemo vs chemo>α-PD-1+α-CTLA-4 groups (**C**), α-PD-1+α-CTLA-4 vs STING/α-PD-1+α-CTLA-4 groups (**D**), chemo>α-PD-1+α-CTLA-4 vs chemo>STING/α-PD-1+α-CTLA-4 group (**E**), chemo>α-PD-1+α-CTLA-4 vs chemo>NLRP3/α-PD-1+α-CTLA-4 groups (**F**), STING/α-PD-1+α-CTLA-4 vs NLRP3/α-PD-1+α-CTLA-4 groups (**G**), STING/α-PD-1+α-CTLA-4 and chemo>STING/α-PD-1+α-CTLA-4 groups (**H**), NLRP3/α-PD-1+α-CTLA-4 and chemo>NLRP3/α-PD-1+α-CTLA-4 groups (**I**), and chemo>STING/α-PD-1+α-CTLA-4 and chemo>NLRP3/α-PD-1+α-CTLA-4 groups (J)

We also performed whole transcriptomic RNA sequencing of tumors harvested from each treatment group in duplicate in the above experiments. We ran unbiased analyses to identify pathways that were differentially expressed with an adjusted p-value (FDR) < 0.05 in tumors from different treatment groups (Fig. 5). We discovered that between the control and chemo group, there was a difference in differentially expressed pathways including the metabolism pathways such as protein digestion and absorption and the tumor-stroma interaction pathways such as ECM-receptor interaction and focal adhesion pathways (Fig. 5A). There were no differentially expressed pathways with FDR < 0.05 comparing between the control and α-PD-1 + α-CTLA-4 groups, a finding consistent with the lack of antitumor response to the α-PD-1 + α-CTLA-4 treatment alone in PDAC. Between α-PD-1 + α-CTLA-4 and chemo > α-PD-1 + α-CTLA-4 groups, we observed differentially expressed pathways including complement and ketone metabolism (Fig. 5B). In the comparison between chemo and chemo > α-PD-1 + α-CTLA-4 groups, we observed differentially expressed pathways including multiple metabolism pathways and tumor-stroma interacting pathways. Such differential expressions appeared to involve more pathways than the comparison between the control and chemo groups, suggesting the α-PD-1 + α-CTLA-4 treatment may further modulate these pathways. Complement, cytokine, and chemokine pathways also appeared to have been modulated by the α-PD-1 + α-CTLA-4 treatment (Fig. 5C). These results suggested that PDAC potentially responds to ICIs immunologically in combination with chemotherapy.

Interestingly, comparing the α-PD-1 + α-CTLA-4 and STING/α-PD-1 + α-CTLA-4 groups, we observed fewer differentially expressed pathways than those observed between the chemo and chemo > α-PD-1 + α-CTLA-4 groups, including multiple metabolism and innate immune response pathways (Fig. 5D). These results suggest that chemotherapy may function as a broader spectrum innate agonist. In contrast, there were no differentially expressed pathways with FDR < 0.05 comparing between the α-PD-1 + α-CTLA-4 and NLRP3/α-PD-1 + α-CTLA-4 groups. Comparing the chemo > α-PD-1 + α-CTLA-4 and chemo > STING/α-PD-1 + α-CTLA-4 groups, the T cell signaling pathway was among the few differentially expressed pathways (Fig. 5E), explaining how adding STING agonist to the chemo > α-PD-1 + α-CTLA-4 treatment potentially may activate effector T cells as seen in the above FACS analysis. Interestingly, comparing between the chemo > α-PD-1 + α-CTLA-4 and chemo > NLRP3/α-PD-1 + α-CTLA-4 groups (Fig. 5F), two metabolism pathways were among the few differentially expressed pathways. Compared between NLRP3/α-PD-1 + α-CTLA-4 and STING/α-PD-1 + α-CTLA-4 groups, the only differentially expressed pathway was the nitrogen metabolism pathway (Fig. 5G). With this data in mind, it would be of future interest to further explore whether the nitrogen metabolism-related pathways mediate the T cell activation by the means of chemo > NLRP3/α-PD-1 + α-CTLA-4 treatment.

Compared between the chemo > STING/α-PD-1 + α-CTLA-4 and STING/α-PD-1 + α-CTLA-4 groups (Fig. 5H), many innate immune response pathways were observed including cytokine and chemokine pathways and TLR, NLR, and CLR pathways. This result suggested that chemotherapy enhances STING agonist-mediated innate immune response. In contrast, compared between the chemo > NLRP3/α-PD-1 + α-CTLA-4 and NLRP3/α-PD-1 + α-CTLA-4 groups (Fig. 5I), T cell, B cell and NK cell signaling pathways were differentially expressed. This result suggested that chemotherapy and NLRP3 in combination with α-PD-1 + α-CTLA-4 can activate the effector cell pathways and can provide a strong activation of adaptive immune responses. Although STING agonist and NLRP3 agonist in combination with α-PD-1 + α-CTLA-4 appeared to be quite different in modulating the signaling pathways, the addition of chemotherapy proved not significant enough to modulate these signaling pathways (Fig. 5J).

### STING agonist alone in combination with ICIs and NLRP3 agonist in combination with chemotherapy upregulate innate immune-related genes at the individual gene level

**Fig. 6:**
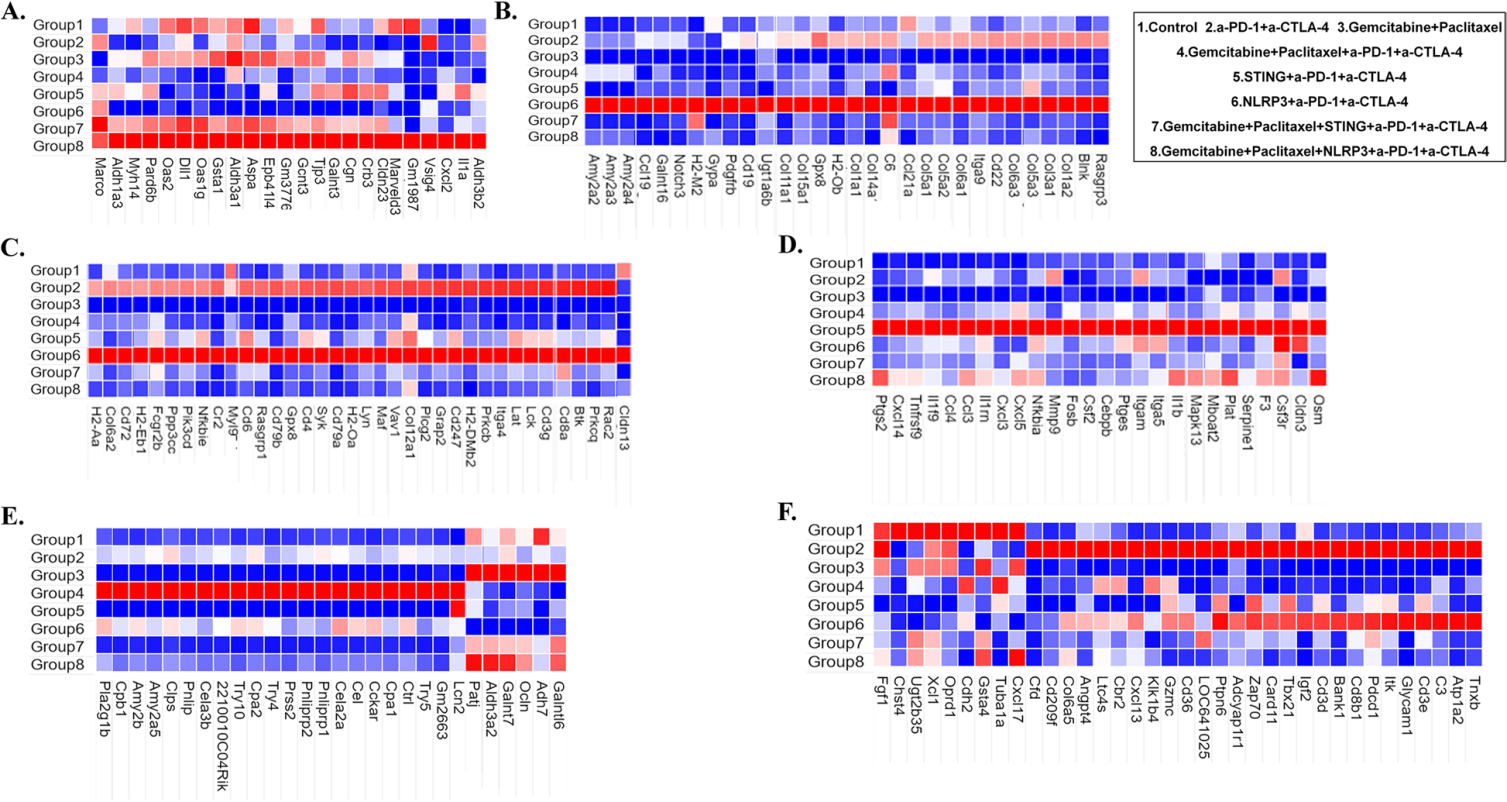
Heatmap of differentially expressed genes of orthotopically implanted tumors between treatment groups. **A** Heatmap shows the most significantly upregulated genes in chemo>STING/α-PD-1+α-CTLA-4 and chemo>NLRP3/α-PD-1+α-CTLA-4 groups. **B**, **C** Heatmap shows the most significantly upregulated genes in NLRP3/α-PD-1+α-CTLA-4 group. **D** Heatmap shows the most significantly upregulated genes in STING/α-PD-1+α-CTLA-4 group. **E** Heatmap shows the most significantly upregulated genes in chemo and chemo>α-PD-1+α-CTLA-4 groups. **F** Heatmap shows the most significantly upregulated genes in control and α-PD-1+α-CTLA-4 groups

We next identified individual genes that were differentially expressed in the tumors in the above comparisons between two treatment groups. Differentially expressed genes with adjusted p-values less than 0.05 were unbiasedly included in the heatmap presented in Fig. 6. We sorted the genes according to their expression levels among the groups in the heap. Interestingly, none of the genes had the highest expression in the chemo > STING/α-PD-1 + α-CTLA-4 group compared to other groups. When we lowered the threshold to a non-adjusted p value at 0.05, we noticed that *Marco*, which encodes a class A scavenger receptor, was the only gene that had higher expression in the chemo > STING/α-PD-1 + α-CTLA-4 group than any other treatment group (Fig. 6A). In contrast, a panel of genes including *Aldh1a3*,* Myh14*,* Pard6b*,* Oas2*,* Dll1*,* Oas1g* had similar expression levels in both the chemo > NLRP3/α-PD-1 + α-CTLA-4 and the chemo > STING/α-PD-1 + α-CTLA-4 groups, but had higher expression levels in these two groups than all other groups (Fig. 6A). Note that *Oas1 and Oas2* encode type I IFN response activated antiviral enzymes. Additional genes including *Gm1987*,* Vsig4*,* Cxcl2*,* IL1a*,* Aldh3b2* had the highest expression in the chemo > NLRP3/α-PD-1 + α-CTLA-4 group, but had a relatively low expression in the chemo > STING/α-PD-1 + α-CTLA-4 group (Fig. 6A). Thus, mechanistically, these genes appear to mediate the effects of the combination of chemotherapy and either innate agonist.

A long list of genes appeared to have the highest expression in the NLRP3/α-PD-1 + α-CTLA-4 group (Fig. 6B and C); however, most of these genes had a similarly high expression in the α-PD-1 + α-CTLA-4 group, suggesting that adding NLRP3 to ICIs may have little additional impacts on the transcriptional regulation (Fig. 6B and C). Interestingly, many of these genes were predominantly fibroblast-associated genes such as the *Fcgr2b* and *Col* genes. In contrast, quite a few genes encoding cytokines and chemokines, or their receptors were exclusively increased in the expression in the STING/α-PD-1 + α-CTLA-4 group (Fig. 6D). In contrast, none of such genes had the highest expression in the Chemo > α-PD-1 + α-CTLA-4 group (Fig. 6E) and *Cxcl13* was the only cytokine gene that had the highest expression in the α-PD-1 + α-CTLA-4 group (Fig. 6F). No immune related genes had the highest expression in either the Chemo alone group or the Control group. These results suggested that STING agonist alone in combination with ICIs upregulates innate immune-related genes at the individual gene level while NLRP3 agonist, with addition of Chemo, upregulates innate immune-related genes.

## Discussion

To our knowledge, this is the first study that has tested the synergistic effect of synthetic innate agonists, ICIs and chemotherapy in animal cancer models. In cancer therapy, the synergistic effect of combining innate immune pathway agonists with ICIs is mechanistically traceable. Previous studies have shown that using vaccines containing STING agonists resulted in significant upregulation of PD-L1 in a mouse model of pancreatic cancer liver metastases, potentially sensitizing pancreatic cancer to ICIs [39]. This was further supported by our other research, where the combination of STING agonists and PD-1 inhibitors demonstrated pronounced immunostimulatory effects, including significant activation of T cell signaling within tumors, but also an upregulation of T cell exhaustion signals. While this combination modestly prolonged survival in a pancreatic cancer liver metastasis model, it was insufficient to elicit durable antitumor responses [21]. To achieve stronger antitumor effects, this study incorporated single agents, double, and triple combinations of synthetic innate agonists including both STING and NLRP3 agonists, chemotherapy, and ICIs in randomized studies in both pancreatic orthotopic syngeneic mouse models and genetically engineered spontaneous tumor models of pancreatic cancer resembling clinical trials of human PDAC patients. These studies demonstrated that antitumor activity of the triple combinations of chemotherapy, innate immune agonists, and ICIs is likely superior over single modalities and double modalities.

Mechanistically, our results highlight that the addition of chemotherapy to either STING or NLRP3 innate agonists enhances the infiltration of effector T cells, especially the memory cytotoxic T cell subtype. Flow cytometry analysis showed a significant increase in CD45 + CD8 + T cells in the TME when chemotherapy was combined with innate agonists and ICIs (Fig. 3A). Further analysis revealed an elevated percentage of CD137 + CD8 + T cells, suggesting heightened T cell activation in response to the combination therapy (Fig. 3B). These data align with previous findings [12] that chemotherapy may offer an in-situ vaccination effect to potentiate the effects of innate immune modulators by inducing immunogenic cell death, thereby facilitating the release of tumor antigens. Interestingly, our study also found distinct differences between STING and NLRP3 agonists in modulating T cell responses. While both agonists enhanced T cell activation and effector memory cell infiltration, the STING agonist exhibited a stronger effect in promoting T cell exhaustion markers such as PD-1 and LAG3 on CD8 + T cells though not significant (Fig. 3G-I). In the tumor-draining lymph nodes, we observed that adding chemotherapy to innate agonists significantly increased the infiltration of CD86 + mature DCs, but not CD40 + DCs (Fig. 4B). This finding indicates that the combined treatment effectively enhances antigen presentation, a critical step for initiating robust T cell responses. Notably, these results are consistent with prior publications demonstrating the central role of CD86 + DCs in mediating antitumor immunity via T cell priming [40]. At the molecular level, RNA sequencing analysis revealed that the combination of chemotherapy and innate agonists upregulated pathways related to T cell activation and nitrogen metabolism (Fig. 5). These findings suggest that nitrogen metabolism may play a role in mediating the enhanced T cell activation observed with NLRP3 agonists, distinguishing their mechanism of action from STING agonists.

The triple combinations of chemotherapy, innate agonist, and ICIs demonstrated the best antitumor activities in every experiment; however, they were not shown to be significantly better compared to double combinations. This limitation could be due to the small sample size and the heterogeneity of spontaneously formed KPC tumors. Despite the breeding challenges of KPC genetically engineered mice and labor-intensiveness of ultrasound measurement, this study holds the largest sample size so far. Nevertheless, the results strongly supported the antitumor efficacy of the triple combination with chemotherapy, innate agonist, and ICIs when compared to chemotherapy alone, thus, providing the rationale for conducting the clinical trials to compare the triple combinations to the standard of care chemotherapy in PDAC patients.

Taken together, the consistent trends in tumor growth suppression, increased TIL infiltration, and enhanced immune signaling strongly support the combination of chemotherapy and innate agonist. This study also showed that the combination of chemotherapy and innate agonist would induce T cell exhaustion and, thus, supported the combination with ICIs or next-generation ICIs such as anti-LAG3 or TIM3 antibodies. This study showed that innate agonists potentiate the role of chemotherapy in inducing DCs, however, fail to induce CD40^+^ DCs. Therefore, it would be interesting to further explore the combination of chemotherapy, a STING or NLRP3 agonist, and a CD40 agonist.

## Conclusions

This study also demonstrated the mechanistic difference between STING and NLRP3 agonists. Although both agonists contribute to the induction of a similar panel of innate immune response genes, STING agonist, but not NLRPS agonist, in combination with chemotherapy appeared to contribute more to the upregulation of T cell activation pathways than chemotherapy. Nevertheless, the combination of NLRP3 agonist and chemotherapy upregulated T cell activation pathways. In addition, this study suggested the role of NLRP3 agonist in modulating the nitrogen metabolism pathways, further distinguishing NLRP3 agonist from STING agonist. Therefore, this study supports a future exploration of the combination of NLRP3 and STING agonist and clinical testing of both STING and NLRP3 agonists, respectively, in combination with chemotherapy in PDAC patients. As our prior study [21] has demonstrated the superiority and feasibility of systemic administration of STING and NLRP3 agonists, treating metastatic PDAC patients with the combination of STING or NLRP3 agonists and chemotherapy has become clinically more feasible.

## Supplementary Information

Supplementary Material 1.

## Data Availability

All data needed to evaluate the conclusions in the paper are present in the paper and the Supplementary Materials. Any further information required to support our data will be supplied upon request.

## Abbreviations

PDAC: Pancreatic ductal adenocarcinoma
ICI: Immune checkpoint inhibitor
TME: Tumor microenvironment
ICD: Immunogenic cell death
STING: Stimulator of Interferon Genes
NLRP3: NLR family pyrin domain containing 3
KPC: LSL-KrasG12D/+; LSL-Trp53R172H/+; Pdx-1-Cre; chemo, gemcitabine plus paclitaxel
α-PD-1: Anti-PD-1 antibody
α-CTLA-4: Anti-CTLA-4 antibody
DCs: Dendritic cells
IP: Intraperitoneal
IM: Intramuscular
IV: Intravenous
PRR: Pattern recognition receptor
RLR: Retinoic acid-inducible gene-I-like receptor
NLR: Nucleotide-binding oligomerization domain-like receptor
TLR: Toll-like receptor
PAMP/DAMP: Pathogen/Damage-associated molecular pattern
CDN: Cyclic dinucleotide
MDSC: Myeloid-derived suppressor cell
Treg: T regulatory cell
IACUC: Institutional Animal Care and Use Committee
SEM: Standard error of the mean
TIL: Tumor infiltrating leukocyte
