# Supplementary material for: Chemotherapy in synergy with innate immune agonists enhances T cell priming for checkpoint inhibitor treatment in pancreatic cancer
Source: Biomark Res. 2025 Jan 27;13:21. doi: 10.1186/s40364-024-00721-7 (PMC11773940; doi:10.1186/s40364-024-00721-7)
Supplement: Supplementary file 1 — Supplementary Material 1. [file 40364_2024_721_MOESM1_ESM.docx]

**Title: Chemotherapy in synergy with innate immune agonists enhances T cell priming for checkpoint inhibitor treatment in pancreatic cancer**

**Supplemental Tables**

**Table S1.** Antibodies for flow cytometry.

**Table S2.** Treatment groups and formula for the KPC spontaneous model.

**Table S3.** Treatment groups and formula for the KPC orthotopic model.

**Supplemental Figures**

**Figure S1.** Individual and overall tumor growth curves in spontaneous KPC mouse model of PDAC with different treatment schema.

**Figure S2.** Kaplan-Meier survival curves compare overall survival between two treatment groups in spontaneous KPC mouse model of PDAC.

**Figure S3.** Individual and overall tumor growth curves in orthotopically implanted KPC tumor model with different treatment schema.

**Figure S4.** Kaplan-Meier survival curves compare overall survival between two different treatment groups in the orthotopically implanted KPC tumor model.

**Figure S5.** Flow cytometry gating strategies.

**Figure S6.** The effects of STING or NLRP3 agonists and their combinations on the

effector T cells in general and memory cytotoxic T cell subtype in the tumors.

**Figure S7.** The effects of STING or NLRP3 agonists and their combinations on the infiltration of effector T cells in general and memory cytotoxic T cell subtype in orthotopically implanted tumor model.

**Figure S8.** The effects of STING or NLRP3 agonists and their based combinations on the induction of DC cells in the tumor draining lymph nodes.

**Table S1 Antibodies for flow cytometry.**

| **Antibodies** | **Manufacturer** | **Catalog #** |
| --- | --- | --- |
| Anti-mouse CD45-PC5.5 | Biolegend | 103132 |
| Anti-mouse CD4-APC/Fire™ 750 | Biolegend | 100568 |
| Anti-mouse CD8-PC7 | Biolegend | 100722 |
| Anti-mouse PD-1-FITC | Biolegend | 135214 |
| Anti-mouse LAG3-PE | Biolegend | 125208 |
| Anti-mouse CD137-APC | Biolegend | 106110 |
| Anti-mouse CD45-APC/Fire™ 750 | Biolegend | 147714 |
| Anti-mouse CD44-FITC | Biolegend | 103005 |
| Anti-mouse CD62L-PE | Biolegend | 104408 |
| Anti-mouse CD11c-APC | Biolegend | 117310 |
| Anti-mouse CD86-PC5.5 | Biolegend | 105028 |
| Anti-mouse CD3e-violet785 | Biolegend | 100355 |
| Anti-mouse CD40-PC7 | Biolegend | 124622 |
| Anti-mouse TIM3-Violet 605^TM^ | Biolegend | 119721 |
| Anti-mouse CCR7-BV421 | BD Biosciences | AB_2737716 |
| Anti-mouse MHC II-FITC | BD Biosciences | AB_394958 |
| Anti-mouse OX40-BV421 | BD Biosciences | AB_2738412 |

**Table S2 Treatment groups and formula for the KPC spontaneous model.**

|  | Reagents | | | | | |
| --- | --- | --- | --- | --- | --- | --- |
| Treatment groups | Week 1 | | Week 2 | | Week 3 | |
|  | Day 4 | Day 7 | Day 11 | Day 14 | Day 18 | Day 21 |
| Control | PBS | PBS |  | |  | |
|  |  | | DPBS+D5W | DPBS+D5W | DPBS+D5W | DPBS+D5W |
|  |  | | α-PD-1+α-CTLA-4 | α-PD-1+α-CTLA-4 | α-PD-1+α-CTLA-4 | α-PD-1+α-CTLA-4 |
| α-PD-1+α-CTLA-4 | PBS | PBS |  | |  | |
|  |  | | DPBS+D5W | DPBS+D5W | DPBS+D5W | DPBS+D5W |
|  |  | | α-PD-1+α-CTLA-4 | α-PD-1+α-CTLA-4 | α-PD-1+α-CTLA-4 | α-PD-1+α-CTLA-4 |
| Chemo | Chemo | Chemo |  | |  | |
|  |  | | DPBS+D5W | DPBS+D5W | DPBS+D5W | DPBS+D5W |
|  |  | | α-PD-1+α-CTLA-4 | α-PD-1+α-CTLA-4 | α-PD-1+α-CTLA-4 | α-PD-1+α-CTLA-4 |
| Chemo>α-PD-1+α-CTLA-4 | Chemo | Chemo |  | |  | |
|  |  | | DPBS+D5W | DPBS+D5W | DPBS+D5W | DPBS+D5W |
|  |  | | α-PD-1+α-CTLA-4 | α-PD-1+α-CTLA-4 | α-PD-1+α-CTLA-4 | α-PD-1+α-CTLA-4 |
| STING/α-PD-1+α-CTLA-4 | PBS | PBS |  | |  | |
|  |  | | STING agonist+D5W | STING agonist+D5W | STING agonist+D5W | STING agonist+D5W |
|  |  | | α-PD-1+α-CTLA-4 | α-PD-1+α-CTLA-4 | α-PD-1+α-CTLA-4 | α-PD-1+α-CTLA-4 |
| NLRP3/α-PD-1+α-CTLA-4 | PBS | PBS |  | |  | |
|  |  | | DPBS+ NLRP3 agonist | DPBS+ NLRP3 agonist | DPBS+ NLRP3 agonist | DPBS+ NLRP3 agonist |
|  |  | | α-PD-1+α-CTLA-4 | α-PD-1+α-CTLA-4 | α-PD-1+α-CTLA-4 | α-PD-1+α-CTLA-4 |
| Chemo>STING+α-PD-1+α-CTLA-4 | Chemo | Chemo |  | |  | |
|  |  | | STING agonist+D5W | STING agonist+D5W | STING agonist+D5W | STING agonist+D5W |
|  |  | | α-PD-1+α-CTLA-4 | α-PD-1+α-CTLA-4 | α-PD-1+α-CTLA-4 | α-PD-1+α-CTLA-4 |
| Chemo>NLRP3+α-PD-1+α-CTLA-4 | Chemo | Chemo |  | |  | |
|  |  | | DPBS+ NLRP3 agonist | DPBS+ NLRP3 agonist | DPBS+ NLRP3 agonist | DPBS+ NLRP3 agonist |
|  |  | | α-PD-1+α-CTLA-4 | α-PD-1+α-CTLA-4 | α-PD-1+α-CTLA-4 | α-PD-1+α-CTLA-4 |
| STING | PBS | PBS |  | |  | |
|  |  | | STING agonist+D5W | STING agonist+D5W | STING agonist+D5W | STING agonist+D5W |
|  |  | | α-PD-1+α-CTLA-4 | α-PD-1+α-CTLA-4 | α-PD-1+α-CTLA-4 | α-PD-1+α-CTLA-4 |
| NLRP3 | PBS | PBS |  | |  | |
|  |  | | DPBS+ NLRP3 agonist | DPBS+ NLRP3 agonist | DPBS+ NLRP3 agonist | DPBS+ NLRP3 agonist |
|  |  | | α-PD-1+α-CTLA-4 | α-PD-1+α-CTLA-4 | α-PD-1+α-CTLA-4 | α-PD-1+MPC-11 |

PBS: vehicle control for chemo; DPBS: vehicle control for STING agonist; D5W: vehicle control for NLRP3 agonist; Chemo: Gemcitabine +Paclitaxel.

**Table S3 Treatment groups and formula for the KPC orthotopic model.**

|  | Reagents | | | | | |
| --- | --- | --- | --- | --- | --- | --- |
| Treatment groups | Week 1 | | Week 2 | | Week 3 | |
|  | Day 4 | Day 7 | Day 11 | Day 14 | Day 18 | Day 21 |
| Control | PBS | PBS |  | |  | |
|  |  | | DPBS+D5W | DPBS+D5W | DPBS+D5W | DPBS+D5W |
|  |  | | α-PD-1+α-CTLA-4 | α-PD-1+α-CTLA-4 | α-PD-1+α-CTLA-4 | α-PD-1+α-CTLA-4 |
| α-PD-1+α-CTLA-4 | PBS | PBS |  | |  | |
|  |  | | DPBS+D5W | DPBS+D5W | DPBS+D5W | DPBS+D5W |
|  |  | | α-PD-1+α-CTLA-4 | α-PD-1+α-CTLA-4 | α-PD-1+α-CTLA-4 | α-PD-1+α-CTLA-4 |
| Chemo | Chemo | Chemo |  | |  | |
|  |  | | DPBS+D5W | DPBS+D5W | DPBS+D5W | DPBS+D5W |
|  |  | | α-PD-1+α-CTLA-4 | α-PD-1+α-CTLA-4 | α-PD-1+α-CTLA-4 | α-PD-1+α-CTLA-4 |
| Chemo>α-PD-1+α-CTLA-4 | Chemo | Chemo |  | |  | |
|  |  | | DPBS+D5W | DPBS+D5W | DPBS+D5W | DPBS+D5W |
|  |  | | α-PD-1+α-CTLA-4 | α-PD-1+α-CTLA-4 | α-PD-1+α-CTLA-4 | α-PD-1+α-CTLA-4 |
| STING/α-PD-1+α-CTLA-4 | PBS | PBS |  | |  | |
|  |  | | STING agonist+D5W | STING agonist+D5W | STING agonist+D5W | STING agonist+D5W |
|  |  | | α-PD-1+α-CTLA-4 | α-PD-1+α-CTLA-4 | α-PD-1+α-CTLA-4 | α-PD-1+α-CTLA-4 |
| NLRP3/α-PD-1+α-CTLA-4 | PBS | PBS |  | |  | |
|  |  | | DPBS+NLRP3 agonist | DPBS+NLRP3 agonist | DPBS+NLRP3 agonist | DPBS+NLRP3 agonist |
|  |  | | α-PD-1+α-CTLA-4 | α-PD-1+α-CTLA-4 | α-PD-1+α-CTLA-4 | α-PD-1+α-CTLA-4 |
| Chemo>STING+α-PD-1+α-CTLA-4 | Chemo | Chemo |  | |  | |
|  |  | | STING agonist+D5W | STING agonist+D5W | STING agonist+D5W | STING agonist+D5W |
|  |  | | α-PD-1+α-CTLA-4 | α-PD-1+α-CTLA-4 | α-PD-1+α-CTLA-4 | α-PD-1+α-CTLA-4 |
| Chemo>NLRP3+α-PD-1+α-CTLA-4 | Chemo | Chemo |  | |  | |
|  |  | | DPBS+NLRP3 agonist | DPBS+NLRP3 agonist | DPBS+NLRP3 agonist | DPBS+NLRP3 agonist |
|  |  | | α-PD-1+α-CTLA-4 | α-PD-1+α-CTLA-4 | α-PD-1+α-CTLA-4 | α-PD-1+α-CTLA-4 |

PBS: vehicle control for chemo; DPBS: vehicle control for STING agonist; D5W: vehicle control for NLRP3 agonist; Chemo: Gemcitabine +Paclitaxel

**
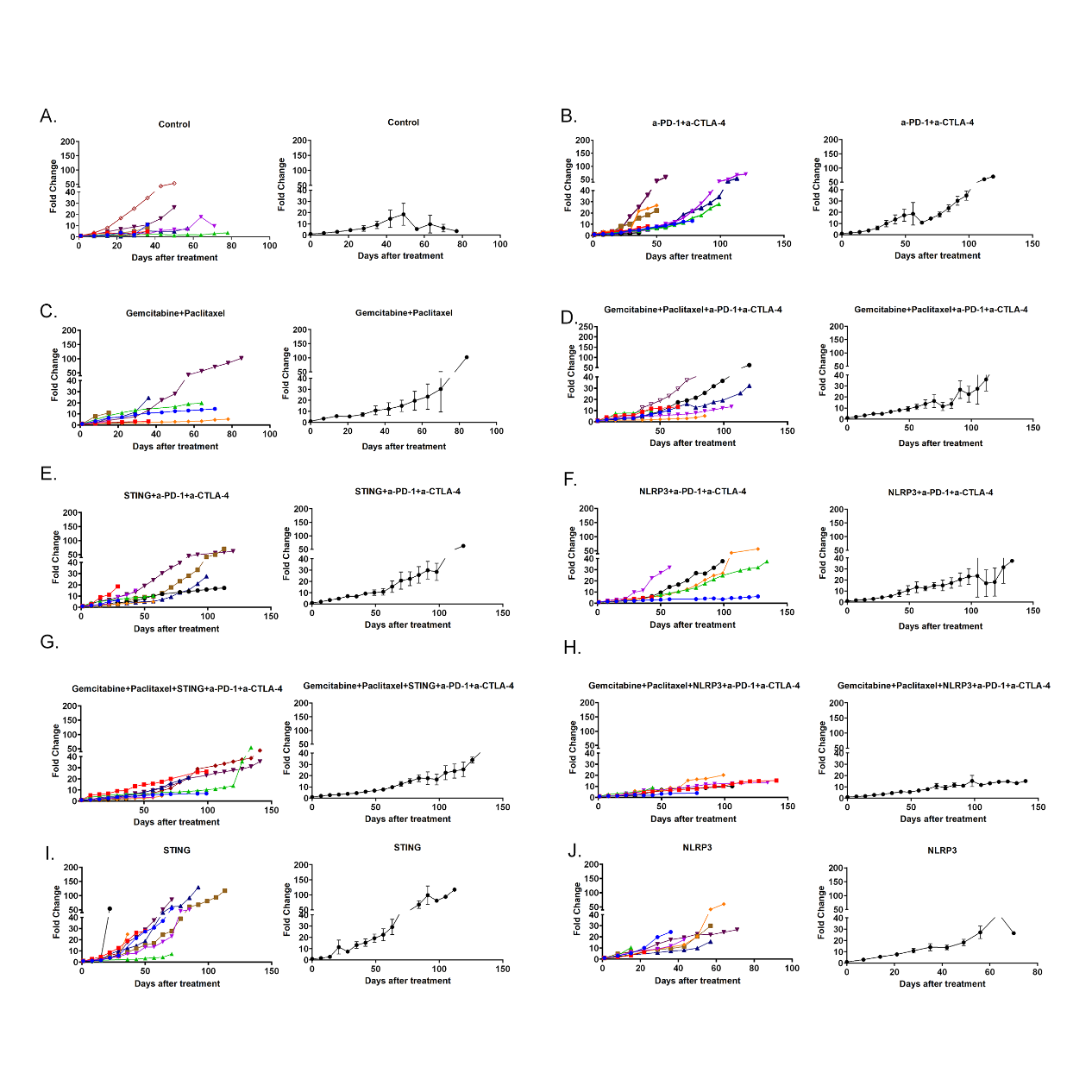
**

**Figure S1. Individual and overall tumor growth curves in spontaneous KPC mouse model of PDAC with different treatment schema.** Tumor volumes were measured once to twice weekly by ultrasound until mice reached the survival endpoint. **(A)** control (n=9), **(B)** α-PD-1+α-CTLA-4 (n=9), **(C)** chemo (n=9), **(D)** chemo>αPD-1+αCTLA-4 (n=8)**, (E)** STING/αPD-1+αCTLA-4 (n=9)**, (F)** NLRP3/αPD-1+αCTLA-4 (n=6)**, (G)** chemo>STING/αPD-1+αCTLA-4 (n=10), **(H)** chemo>NLRP3/αPD-1+αCTLA-4 (n=6), **(I)** STING (n=10), and **(J)** NLRP3 (n=9) treatments. In each panel, left showed growth curves of individual mice within the same treatment group; right showed curves of mean ± SEM.

**
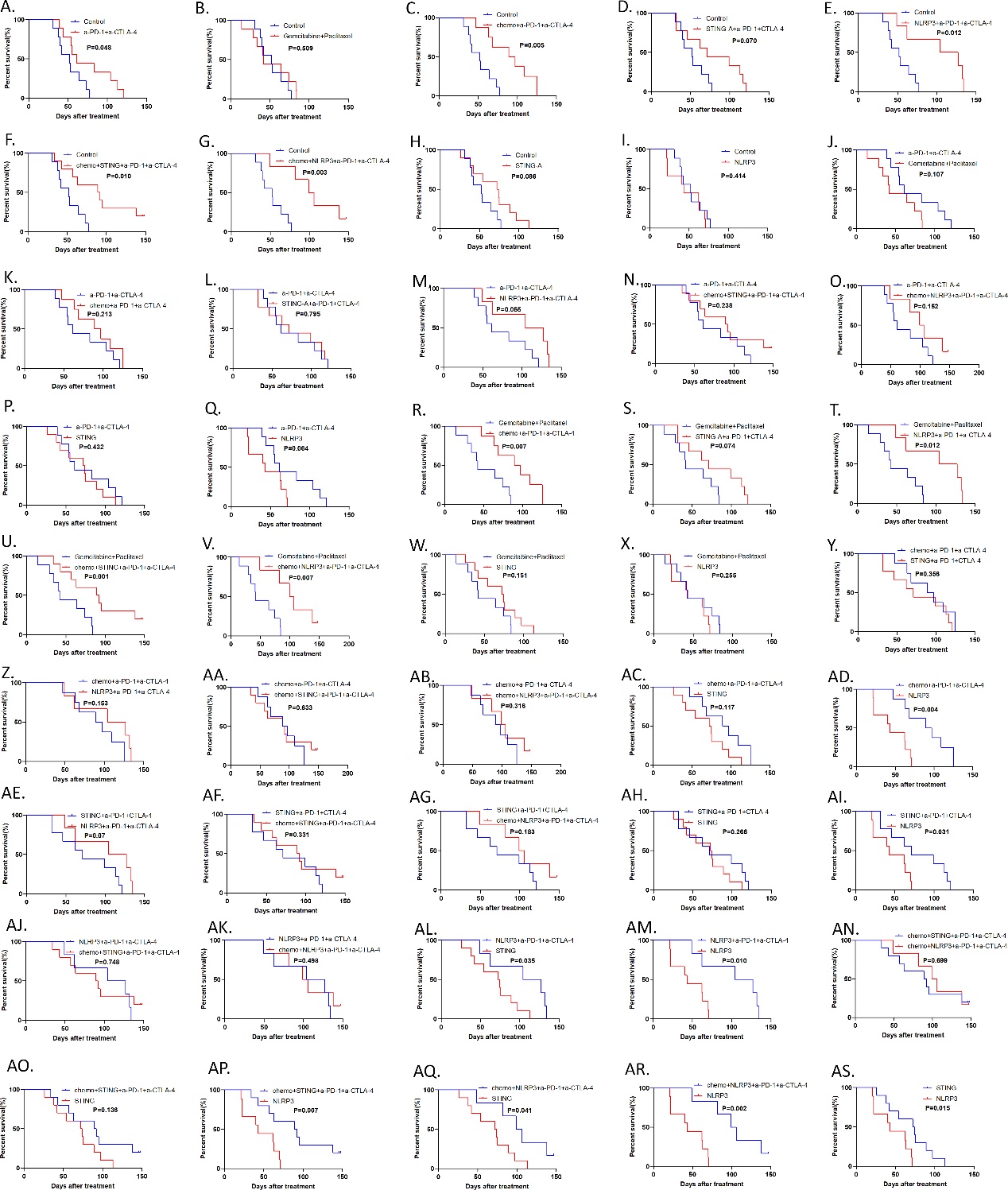
**

**Figure S2. Kaplan-Meier survival curves compare overall survival between two treatment groups in spontaneous KPC mouse model of PDAC. (A)** control vs αPD-1+αCTLA-4**, (B)** control vs chemo, **(C)** control vs chemo>αPD-1+αCTLA-4, **(D)** control vs STING/αPD-1+αCTLA-4**, (E)** control vs NLRP3/αPD-1+αCTLA-4, **(F)** control vs chemo>STING/αPD-1+αCTLA-4, **(G)** control vs chemo>NLRP3/αPD-1+αCTLA-4, **(H)** control vs STING, **(I)** control vs NLRP3, **(J)** αPD-1+αCTLA-4 vs chemo, **(K)** αPD-1+αCTLA-4 vs chemo>αPD-1+αCTLA-4, **(L)** αPD-1+αCTLA-4 vs STING/αPD-1+αCTLA-4**, (M)** αPD-1+αCTLA-4 vs NLRP3/αPD-1+αCTLA-4, **(N)** αPD-1+αCTLA-4 vs chemo>STING/αPD-1+αCTLA-4, **(O)** αPD-1+αCTLA-4 vs chemo>NLRP3/αPD-1+αCTLA-4, **(P)** αPD-1+αCTLA-4 vs STING, **(Q)** αPD-1+αCTLA-4 vs NLRP3, **(R)** chemo vs chemo>αPD-1+αCTLA-4, **(S)** chemo vs STING/αPD-1+αCTLA-4**, (T)** chemo vs NLRP3/αPD-1+αCTLA-4, **(U)** chemo vs chemo>STING/αPD-1+αCTLA-4, **(V)** chemo vs chemo>NLRP3/αPD-1+αCTLA-4, **(W)** chemo vs STING, **(X)** chemo vs NLRP3, **(Y)** chemo>αPD-1+αCTLA-4 vs STING/αPD-1+αCTLA-4**, (Z)** chemo>αPD-1+αCTLA-4 vs NLRP3/αPD-1+αCTLA-4, **(AA)** chemo>αPD-1+αCTLA-4 vs chemo>STING/αPD-1+αCTLA-4, **(AB)** chemo>αPD-1+αCTLA-4 vs chemo>NLRP3/αPD-1+αCTLA-4, **(AC)** chemo>αPD-1+αCTLA-4 vs STING, **(AD)** chemo>αPD-1+αCTLA-4 vs NLRP3, **(AE)** STING/αPD-1+αCTLA-4 vs NLRP3/αPD-1+αCTLA-4, **(AF)** STING/αPD-1+αCTLA-4 vs chemo>STING/αPD-1+αCTLA-4, **(AG)** STING/αPD-1+αCTLA-4 vs chemo>NLRP3/αPD-1+αCTLA-4, **(AH)** STING/αPD-1+αCTLA-4 vs STING, **(AI)** STING/αPD-1+αCTLA-4 vs NLRP3, **(AJ)** NLRP3/αPD-1+αCTLA-4 vs chemo>STING/αPD-1+αCTLA-4, **(AK)** NLRP3/αPD-1+αCTLA-4 vs chemo>NLRP3/αPD-1+αCTLA-4, **(AL)** NLRP3/αPD-1+αCTLA-4 vs STING, **(AM)** NLRP3/αPD-1+αCTLA-4 vs NLRP3, **(AN)** chemo>STING/αPD-1+αCTLA-4 vs chemo>NLRP3/αPD-1+αCTLA-4, **(AO)** chemo>STING/αPD-1+αCTLA-4 vs STING, **(AP)** chemo>STING/αPD-1+αCTLA-4 vs NLRP3, **(AQ)** chemo>STING/αPD-1+αCTLA-4 vs STING, **(AR)** chemo>STING/αPD-1+αCTLA-4 vs NLRP3, and **(AS)** STING vs NLRP3 treatments. P-value less than 0.05 was considered statistically significant.

**
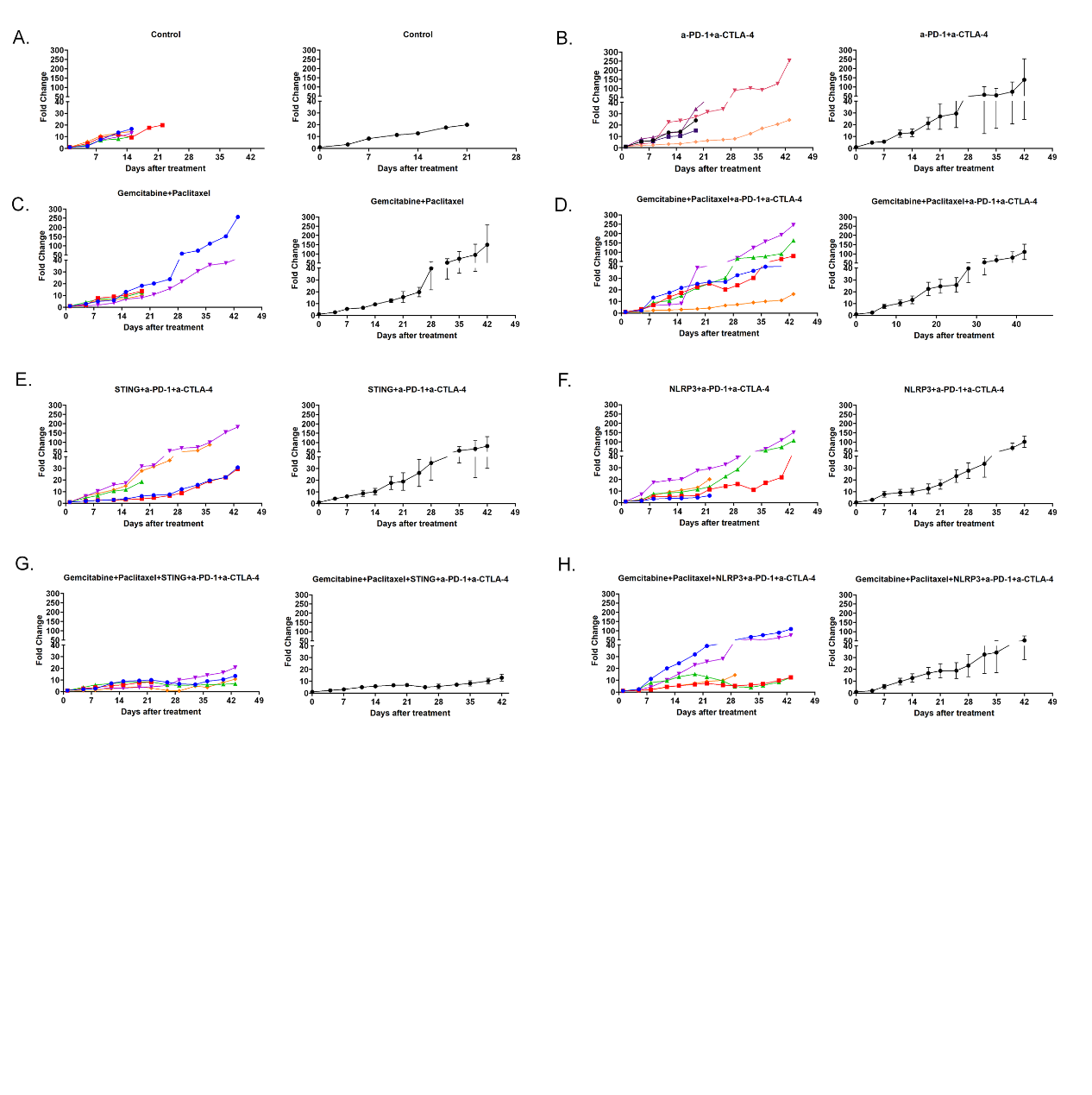
**

**Figure S3. Individual and overall tumor growth curves in orthotopically implanted KPC tumor model with different treatment schema.** Tumor volumes were measured once to twice weekly by ultrasound until mice reached the survival endpoint. **(A)** control, **(B)** α-PD-1+α-CTLA-4, **(C)** chemo, **(D)** chemo>αPD-1+αCTLA-4, **(E)** STING/αPD-1+αCTLA-4, **(F)** NLRP3/αPD-1+αCTLA-4, **(G)** chemo>STING/αPD-1+αCTLA-4, and **(H)** chemo>NLRP3/αPD-1+αCTLA-4 treatments (n=5 per group). In each panel, left showed growth curves of individual mice within the same treatment group; right showed curves of mean ± SEM.

**
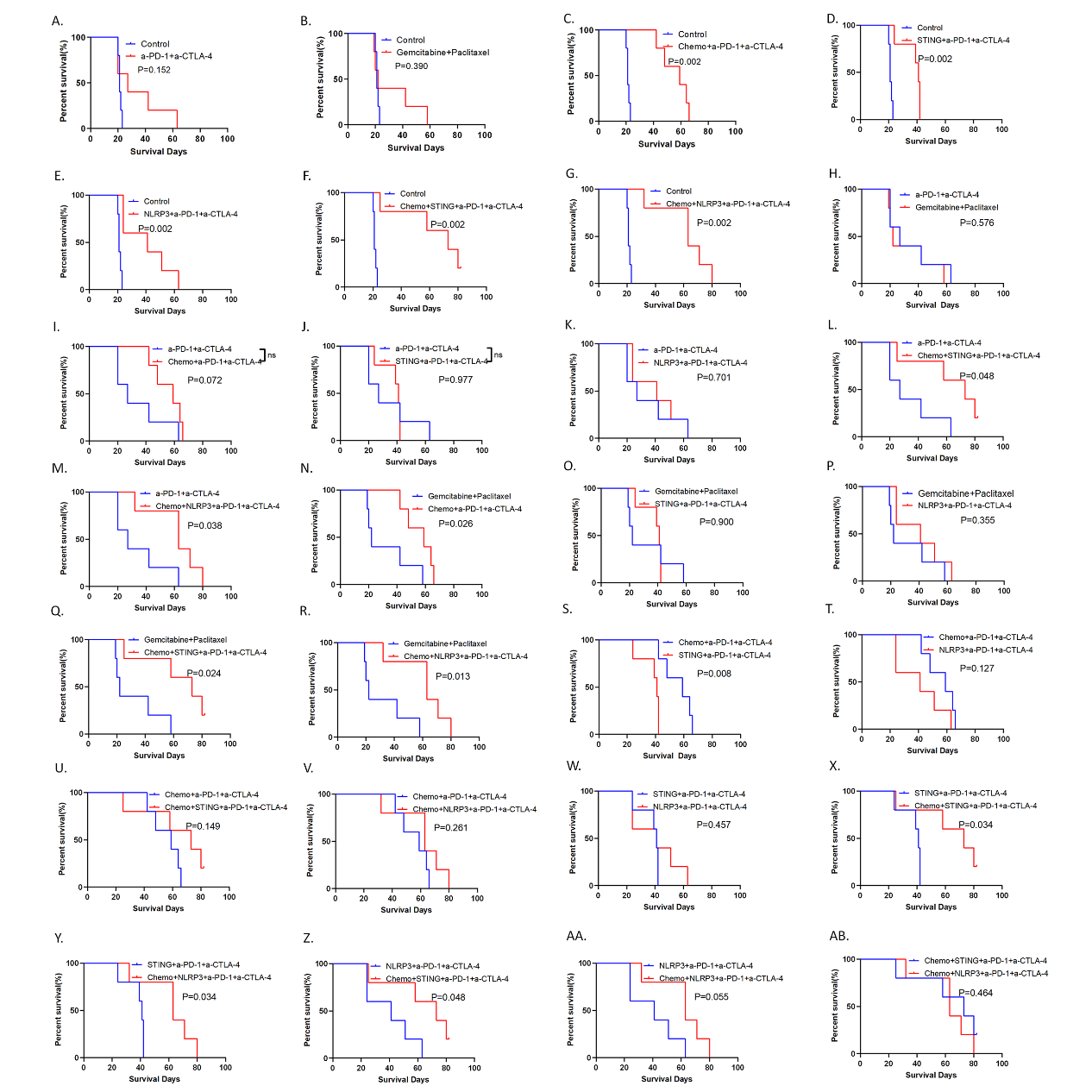
**

**Figure S4. Kaplan-Meier survival curves compare overall survival between two different treatment groups in the orthotopically implanted KPC tumor model. (A)** control vs αPD-1+αCTLA-4**, (B)** control vs chemo, **(C)** control vs chemo>αPD-1+αCTLA-4, **(D)** control vs STING/αPD-1+αCTLA-4**, (E)** control vs NLRP3/αPD-1+αCTLA-4, **(F)** control vs chemo>STING/αPD-1+αCTLA-4, **(G)** control vs chemo>NLRP3/αPD-1+αCTLA-4, **(H)** αPD-1+αCTLA-4 vs chemo, **(I)** αPD-1+αCTLA-4 vs chemo>αPD-1+αCTLA-4, **(J)** αPD-1+αCTLA-4 vs STING/αPD-1+αCTLA-4**, (K)** αPD-1+αCTLA-4 vs NLRP3/αPD-1+αCTLA-4, **(L)** αPD-1+αCTLA-4 vs chemo>STING/αPD-1+αCTLA-4, **(M)** αPD-1+αCTLA-4 vs chemo>NLRP3/αPD-1+αCTLA-4, **(N)** chemo vs chemo>αPD-1+αCTLA-4, **(O)** chemo vs STING/αPD-1+αCTLA-4**, (P)** chemo vs NLRP3/αPD-1+αCTLA-4, **(Q)** chemo vs chemo>STING/αPD-1+αCTLA-4, **(R)** chemo vs chemo>NLRP3/αPD-1+αCTLA-4, **(S)** chemo>αPD-1+αCTLA-4 vs STING/αPD-1+αCTLA-4**, (T)** chemo>αPD-1+αCTLA-4 vs NLRP3/αPD-1+αCTLA-4, **(U)** chemo>αPD-1+αCTLA-4 vs chemo>STING/αPD-1+αCTLA-4, **(V)** chemo>αPD-1+αCTLA-4 vs chemo>NLRP3/αPD-1+αCTLA-4, **(W)** STING/αPD-1+αCTLA-4 vs NLRP3/αPD-1+αCTLA-4, **(X)** STING/αPD-1+αCTLA-4 vs chemo>STING/αPD-1+αCTLA-4, **(Y)** STING/αPD-1+αCTLA-4 vs chemo>NLRP3/αPD-1+αCTLA-4, **(Z)** NLRP3/αPD-1+αCTLA-4 vs chemo>STING/αPD-1+αCTLA-4, **(AA)** NLRP3/αPD-1+αCTLA-4 vs chemo>NLRP3/αPD-1+αCTLA-4, and **(AB)** chemo>STING/αPD-1+αCTLA-4 vs chemo>NLRP3/αPD-1+αCTLA-4 treatments. P-value less than 0.05 was considered statistically significant.

**
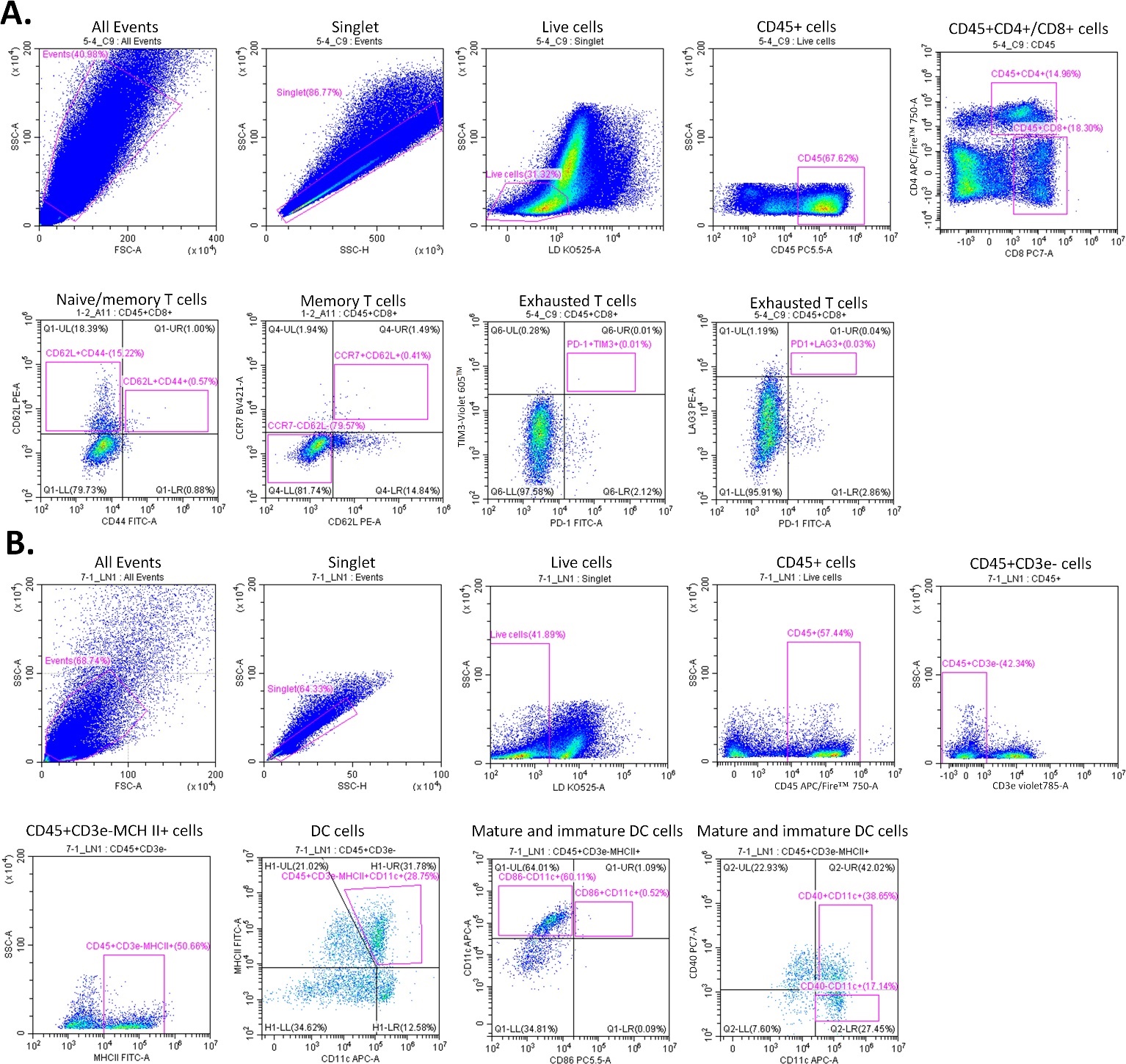
**

**Figure S5. Flow cytometry gating strategies.** **(A)** Flow cytometry gating strategy for identification of naive/central memory/effector/Memory/exhausted T cells. First, side scatter height (SSC-H) and side scatter area (SSC-A) plots were used to exclude doublets. Dead cells were excluded by gating on cells negative for the viability marker Aqua Blue. TIM3 and LAG3 was used to identify exhausted T cells (CD8^+^TIM3^+^, CD8^+^LAG3^+^) T cells. Exhausted T cells were further defined as CD8^+^PD-1^+^TIM3^+^, CD8^+^PD-1^+^LAG3^+^). CD44 was used to distinguish between naive (CD8^+^CD44^−^) and memory (CD8^+^CD44^+^) T cells. Naive T cells were defined as CD8^+^CD62L^+^CD44^−^. CD62L and CCR7 was used to define central memory T cells (CD8^+^CD62L^+^CCR7^+^) and effector memory T cells (CD8^+^ CD62L^−^CCR7^−^). **(B)** Flow cytometry gating strategy for DC cells is shown. DC cells were identified based on their forward- and side- scatter properties. Subsequently, singlet cells were gated; and dead cells were excluded by gating on cells negative for the viability marker Aqua Blue. CD40 was used to distinguish between mature (CD3e^−^MHCII^+^CD40^+^) and immature (CD3e^−^MHCII^+^CD40-) DCs. CD86 was also used to distinguish between mature (CD3e^−^MHCII^+^CD86^+^) and immature (CD3e^−^MHCII^+^CD86^−^) DC cells.

**
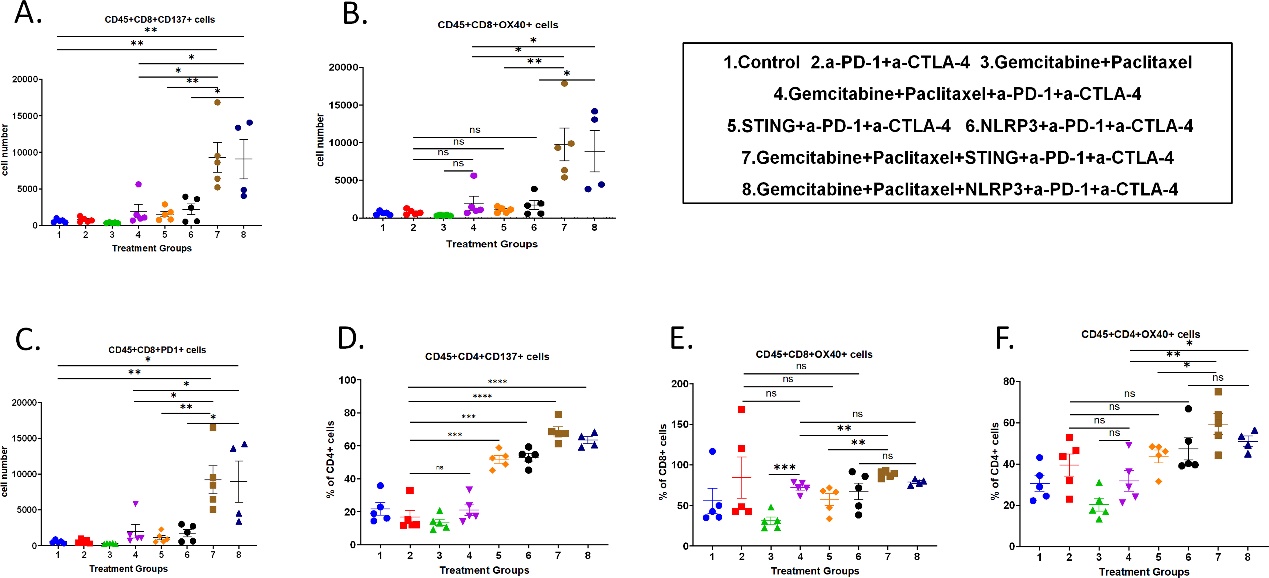
**

**Figure S6. The effects of STING or NLRP3 agonists and their combinations on the**

**effector T cells in general and memory cytotoxic T cell subtype in the tumors. (A-C)** Cell number of the CD137^+^CD8^+^ T cells **(A)**, OX40^+^CD8^+^ T cells **(B)**, and PD-1^+^CD8^+^ T cells **(C)** in the tumors. **(D)** Percentages of the CD137^+^CD4^+^ T cells among CD4+ T cells in the tumors. **(E)** Percentages of the OX40^+^CD8^+^ T cells among CD8^+^ T cells in the tumors. **(F)** Percentages of the OX40^+^CD4^+^ T cells among CD4^+^ T cells in the tumors (n=5 per group except n=4 in chemo>NLRP3/α-PD-1+α-CTLA-4). Data shown as mean ± SEM; compared by unpaired t test; *p<0.05; **p<0.01; ***p<0.001; ****p<0.0001. NS, not significant.


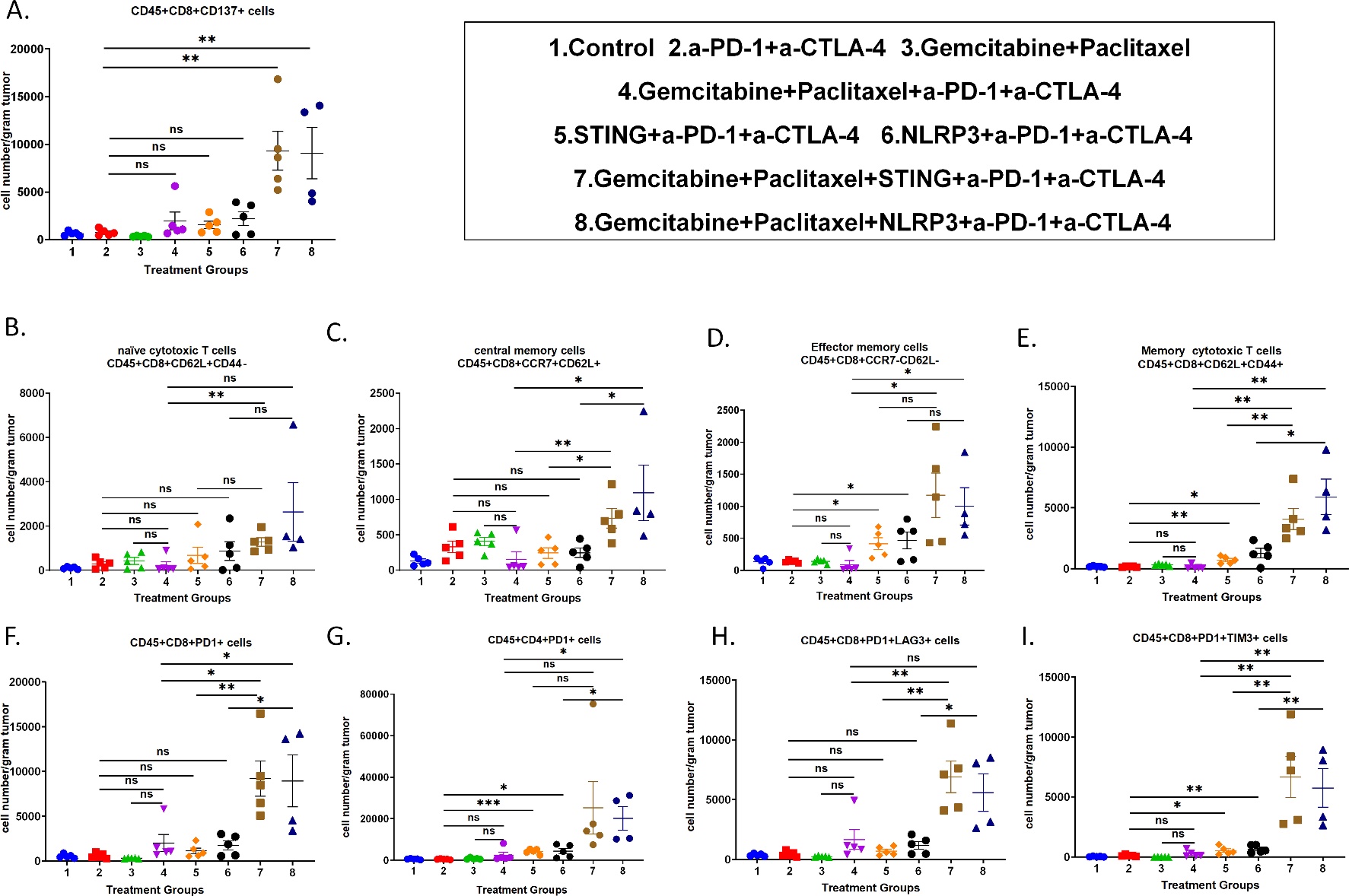


**Figure S7. The effects of STING or NLRP3 agonists and their combinations on the infiltration of effector T cells in general and memory cytotoxic T cell subtype in orthotopically implanted tumor model.** Cell number of CD137^+^CD8^+^ T cells **(A)**, CD62^+^CD44^-^ naïve cytotoxic T cells **(B)**, CCR7^+^CD62L^+^ central memory T cells **(C)**, CCR7^-^CD62L^-^ effector memory T cells **(D)**, CD62L^+^CD44^+^ memory cytotoxic T cells **(E)**, PD-1^+^CD8^+^ T cells **(F)**, PD-1^+^CD4^+^ T cells **(G),** PD-1^+^LAG3^+^CD8^+^ T cells**(H),** PD-1^+^TIM3^+^CD8^+^ T cells **(I)** in the tumors normalized by tumor weight per gram (n=5 per group except n=4 in chemo>NLRP3/α-PD-1+α-CTLA-4). Data shown as mean ± SEM and compared by unpaired t test; *p<0.05; **p<0.01. NS, not significant.

**
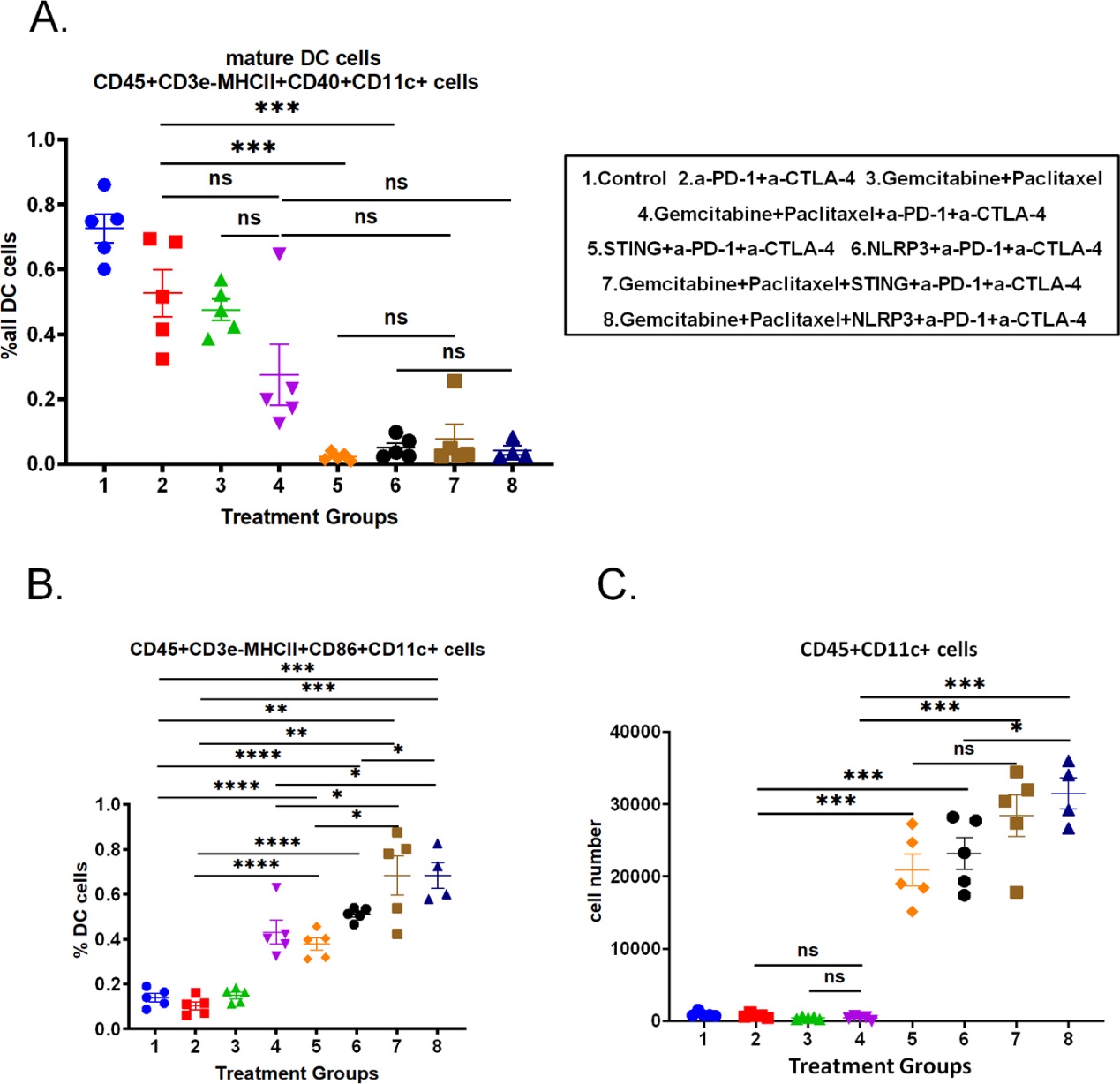
**

**Figure S8. The effects of STING or NLRP3 agonists and their based combinations on the induction of DC cells in the tumor draining lymph nodes. (A-B)** Percentages of CD40^+^ DC **(A),** and CD86^+^ DC **(B)** among all DC cells. **(C)** Number of CD45^+^CD11c^+^ myeloid cells in the tumor draining lymph nodes (n=5 per group except n=4 in chemo>NLRP3/α-PD-1+α-CTLA-4). Data shown as mean ± SEM; compared by unpaired t test; *p<0.05; **p<0.01; ***p<0.001; ****p<0.0001. NS, not significant.
